# Integrating tick density and park visitor behaviors to assess the risk of tick exposure in urban parks on Staten Island, New York

**DOI:** 10.1186/s12889-022-13989-x

**Published:** 2022-08-23

**Authors:** Erin Hassett, Maria Diuk-Wasser, Laura Harrington, Pilar Fernandez

**Affiliations:** 1 Department of Entomology, Cornell University, Ithaca, NY 14850 USA; 2 Department of Environmental Science, SUNY College of Environmental Science and Forestry, Syracuse, NY 13210 USA; 3 Department of Ecology, Evolution, and Environmental Biology, Columbia University, New York, NY 10027 USA; 4 Paul G. Allen School for Global Health, Washington State University, Pullman, WA 99164 USA

**Keywords:** Tick-borne diseases, Ixodes scapularis, Lyme disease, Park usage

## Abstract

**Background:**

Public green spaces are important for human health, but they may expose visitors to ticks and tick-borne pathogens. We sought to understand, for the first time, visitors’ exposure risk and drivers of tick-preventative behavior in three popular parks on Staten Island, New York City, NY, USA, by integrating tick hazard and park visitors’ behaviors, risk perceptions and knowledge.

**Methods:**

We conducted tick sampling in three parks, across three site types (open spaces, the edge of open spaces, and trails) and three within-park habitats (maintained grass, unmaintained herbaceous, and leaf litter) to estimate tick density during May-August 2019. Human behavior was assessed by observations of time spent and activity type in each site. We integrated the time spent in each location by park visitors and the tick density to estimate the probability of human-tick encounter. To assess visitors’ tick prevention behaviors, a knowledge, attitude, and practices (KAP) survey was administered.

**Results:**

Three tick species (*Ixodes scapularis*, *Amblyomma americanum* and *Haemaphysalis longicornis)* were collected. For all species, the density of nymphs was greatest in unmaintained herbaceous habitats and trails, however, the fewest people entered these hazardous locations. The KAP survey revealed that most respondents (*N* = 190) identified parks as the main location for tick exposure, but most believed they had minimal risk for tick encounter. Consequently, many visitors did not conduct tick checks. People were most likely to practice tick checks if they knew multiple prevention methods and perceived a high likelihood of tick encounter.

**Conclusions:**

By integrating acarological indices with park visitor behaviors, we found a mismatch between areas with higher tick densities and areas more frequently used by park visitors. However, this exposure risk varied among demographic groups, the type of activities and parks, with a higher probability of human-tick encounters in trails compared to open spaces. Furthermore, we showed that people’s KAP did not change across parks even if parks represented different exposure risks. Our research is a first step towards identifying visitor risk, attitudes, and practices that could be targeted by optimized messaging strategies for tick bite prevention among park visitors.

**Supplementary Information:**

The online version contains supplementary material available at 10.1186/s12889-022-13989-x.

## Introduction

Vector-borne diseases are an increasing public health challenge in the United States, with tick-borne diseases accounting for the majority (77%) of all cases reported in the last decade, of which more than 80% were Lyme disease cases [1]. An estimated 476,000 cases of Lyme disease occur in the United States each year, predominately in the Northeast, Mid-Atlantic, and Upper-Midwest regions [2]. The epidemiology of Lyme disease is complex, and *Borrelia burgdorferi* sensu stricto (the primary Lyme disease-causing bacterium in North America) is maintained in enzootic transmission cycles between *Ixodes scapularis* ticks and multiple hosts; humans are considered incidental hosts. Thus, Lyme disease risk depends on both the density of infected ticks, typically nymphs (hereafter, tick hazard) and on human behaviors affecting exposure (e.g. avoidance behaviors and use of personal protective measures) [3].

Human exposure to *I. scapularis* ticks, the vector of *B. burgdorferi* in eastern United States, occurs outdoors in proximity to natural or peridomestic wooded settings. However, while links between tick density in residential yards and human disease have been extensively documented [4–10], there is little and only passive information on what proportion of infectious tick exposures occur in parks [11, 12]. Furthermore, other tick species are expanding in distribution, such as *Amblyomma americanum,* an aggressive human biter that transmits *Ehrlichia chaffeensis, Francisella tularensis,* Heartland Virus, and *Haemaphysalis longicornis,* the more newly invasive tick that transmits Severe Fever with Thrombocytopenia Syndrome Virus in its native range [13, 14]. Elucidating tick encounter risk to these additional tick species in parks is important to understand visitor exposure to new regional pathogens and if concurrent exposure of humans to these additional tick species with different questing behaviors, can prompt changes in human behavior (i.e., adaptive responses) that can impact human-*Ixodes* encounter rates.

Estimating tick encounters in public green spaces, including neighborhoods, states, and national parks presents many challenges. Human exposure risk to ticks and pathogens has been previously estimated in public green spaces by using a drag cloth sampling technique and determining the infected tick encounter rate per hour [15] or tick encounter distance (number of meters passed until encountering a tick) on frequently used trails [16]. However, these studies only use acarological measures and do not examine human usage (e.g., time spent in different park areas and habitats) to assess exposure. Moreover, tick density and pathogen distribution can vary widely between and within parks in the same area [17–19], resulting in spatial heterogeneities in the tick hazard. To the best of our knowledge, our study is the first to simultaneously (in time and space) assess tick hazard and park users’ behaviors to determine the baseline risk of human-tick encounters in public parks.

Urban green spaces provide many ecosystem services to humans “to sustain or enhance health and well-being” [20]. The importance of green spaces for human well-being has been extensively studied, particularly during the COVID-19 pandemic, and reported benefits include stress reduction [21], mental fatigue relief [22, 23], violence reduction [24], and increased sense of happiness [25]. However, there is increasing concern about growing health threats from tick-borne diseases in and around urban environments since green spaces, such as parks within urban centers, can provide suitable habitat for ticks infected with various tick-borne pathogens [26, 27]. Nonetheless, the majority of tick-borne disease studies have been conducted in areas of low intensity residential developments and in natural areas. In this study, we focus on the risk of human exposure to ticks in an urban area with high population density and extensive park systems (Staten Island, New York City, NY), where locally acquired tick-borne diseases have been on the rise [28].

A fundamental challenge in understanding people’s risk of exposure to ticks in multi-use urban parks is the extremely high heterogeneity in the time people spend and activities performed in habitats with highly contrasting tick exposure hazard. While research in mosquito-borne pathogens has long acknowledged the importance of incorporating human movement and habitat use into risk assessments [29, 30], this integration has not been previously attempted for tick-borne diseases. From an individual perspective, a person’s risk of exposure to a vector can be represented by an exposure model that depends on vector abundance, the biting rate of the vector, the time spent at a given location where vectors are present and the cumulative probability of an encounter given the time spent [29]. This individual exposure risk can only be estimated by following individuals in their daily activities. However, the cumulative probability of human-vector encounter (i.e., how ‘risky’ a site is) can be used to compare the risk of vector exposure across sites with different vector densities and use patterns by humans. Herein, we present a novel index for the risk of human exposure to three important tick vectors (i.e., *I. scapularis, A. americanum,* and *H. longicornis*) [19, 31] by estimating the tick hazard (density of nymphal stages in frequently visited areas of parks), assessing the length of time spent in tick habitat and type of activities performed by park visitors, and calculating the probability of human-tick encounters per site and park. This index can be used to compare the risk of vector exposure across sites with different vector densities and use patterns by humans, thus representing a useful management tool.

The probability of human-tick encounter would be further modified by visitors’ past experiences with ticks and tick-borne diseases, and their perceived tick risk and knowledge may influence their use of protective measures to minimize tick bites. Knowledge, attitude, and practice (KAP) surveys are ideal tools for identifying gaps in the public’s knowledge and response to tick exposure risk. Current KAP research demonstrates that respondents minimally and irregularly exercise tick preventative practices [32, 33]. Additionally, predictors of practicing tick preventative measures vary by study and are influenced by respondents’ prior experience, knowledge about and attitude towards the topic [32–34]. To understand how individuals visiting urban parks are motivated to practice tick preventative behaviors, we performed KAP assessments in situ.

In this study, we uniquely assessed estimates of tick encounter risk by park visitors based on observations of human activity (exposure time in different park sites and habitats) and tick abundance in those same areas. We determined tick encounter risk and then administered KAP surveys to the park visitor population to assess visitor responses to tick and tick-borne pathogen exposure risk. Our results can provide a basis for optimizing tick bite prevention and outreach measures to protect human health in urban park settings.

## Materials and methods

### Field sites

Staten Island (Richmond County) is one of five boroughs in New York City (NYC), with an estimated population of 476,000 people as of 2018 [35, 36]. The island is composed of neighborhoods exhibiting differences in housing structure and demographic and socioeconomic composition; overall, 75.2% of the population identifies as White or Caucasian, 11.7% Black or African American, 10.2% Asian, and 18.7% identifies as Hispanic or Latino [37]. Known as the “borough-of-parks”, 18% of the total area is covered by urban parks and forests [35], and an assessment of tick populations in NYC showed that most tick species were established on Staten Island and only a few focal areas in the Bronx borough [38]. The rate of locally-acquired Lyme disease cases on Staten Island has increased from 4 to 25 per 100,000 between 2000 and 2016 [39].

**Fig. 1:**
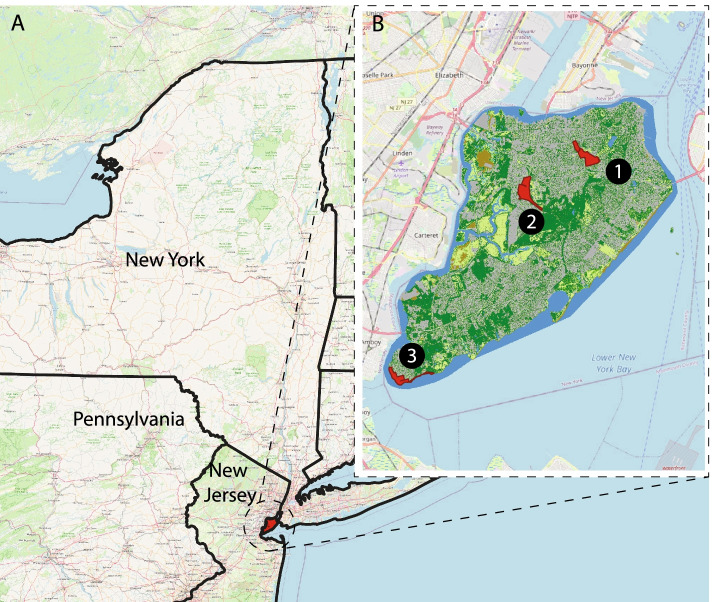
Staten Island, NY (panel **A**) and location of study parks (panel **B**): 1 Clove Lakes; 2 Willowbrook; 3 Conference House

Three public parks were selected (see Fig. 1): Clove Lakes Park (40°37′07.2″N 74°06′41.7″W), Willowbrook Park (40°36′03.2″N 74°09′29.9″W), and Conference House Park (40°30′02.1″N 74°15′04.4″W). These parks were selected due to their previously observed high volume of park visitors and a range in tick density, with the lowest density of ticks at Clove Lakes in the north and the highest density of ticks at Conference House in the south [19, 31]. Open spaces and hiking trails were selected in each of the parks to assess human use and tick density, and these areas were selected based on site availability in the park (smaller parks had fewer open areas and trails) and presence of potential tick habitat (Additional file 1). We selected 14 sites in total: 6 sites in Clove Lakes, 4 sites in Willowbrook and 4 sites in Conference House, with 1:1 distribution between open spaces and trails. Prior to conducting tick sampling and park use assessments, we defined the boundaries of the open areas by determining the field of view from various points and identifying landmarks that could act as limits (i.e., paths, woodlines, and waterlines). For hiking trails, we identified entry/exit points or intersections with high pedestrian traffic.

### Tick collections

From 20 May to 19 August 2019, we collected questing ticks using a 1 m^2^ white corduroy cloth (tick drag). Depending on the size of the habitat available per site, transects of 100 m were sampled, otherwise shorter transects were sampled. Attached ticks were removed every 20 m along the length of every transect [19, 31, 40]. Ticks were placed in 70% ethanol and later identified to species and sex using a dissecting microscope and appropriate taxonomic keys [41]. Drag lengths were measured using BasicAirData GPS Logger ver. 2.2.4 app for Android and GPS Tracker Pro app for iPhone 6 s, and lengths were verified using the program Garmin BaseCamp 4.8.3.

The 14 sites (Additional file 1) were sampled once a week between 8 AM and 7 PM, and during each visit, at least three drag samples were collected at each site. In open areas, drags were performed at the edges of the open area and within the open area (mowed lawn space). These edges often consisted of strips of vegetation along wood margins, waterlines, or natural paths. Impervious surfaces such as paved paths were excluded from sampling. For trail transects, drags were performed on the sides of the trail within the vegetation, and a 10 m buffer was kept between consecutive transects. Drags were not performed if vegetation was wet. Sampling sites were restricted to areas park visitors frequent to gauge risk for tick interaction (public trails or open lawn spaces, excluding inaccessible forested areas) and corresponded to the areas where human behavioral observations were performed. At Clove Lakes, two trails were unavailable for sampling after 21 July due to construction, vegetation removal, and inaccessibility.

### Habitat classification

At each site, habitats were classified into five categories: maintained grass (regularly mowed lawn), unmaintained herbaceous, leaf litter, impervious, and edge (Additional file 2). Edge habitats (i.e., vegetation bordering an open area) consisted of unmaintained herbaceous or leaf litter habitats and occurred between 1) impervious and forest (i.e., strip of vegetation next to paved paths at the limit of the open area and the forest), 2) maintained grass and forest, and 3) maintained grass and water (i.e., brush/natural path at the waterline). Varying numbers of drags were performed in each site type and habitat, depending on habitat availability in each park (Additional file 3). Vegetation in unmaintained herbaceous and leaf litter habitats included a variety of species (Additional file 4). Maintained grass habitats comprised various graminoid species.

### Park visitor observations

From 20 May to 19 August 2019, park usage by visitors was determined by observing each park site for 30 min within set time intervals: 9 am-12 pm, 12 pm-3 pm, 3 pm-6 pm (adapted from ﻿Goličnik & Ward Thompson) [42]. During these 14 weeks, we conducted observations Monday through Sunday at the three time intervals specified above (i.e., morning, afternoon, and evening). We collected park usage data at least two times for each day of the week (7) and each time interval (3) during the study period, reaching a minimum of 30-min observations performed for each of the 14 sites (294 observation hours total). In circumstances when observations for each site were incomplete (e.g., due to weather conditions), sites were observed again later in the season for the respective missing interval.

**Fig. 2:**
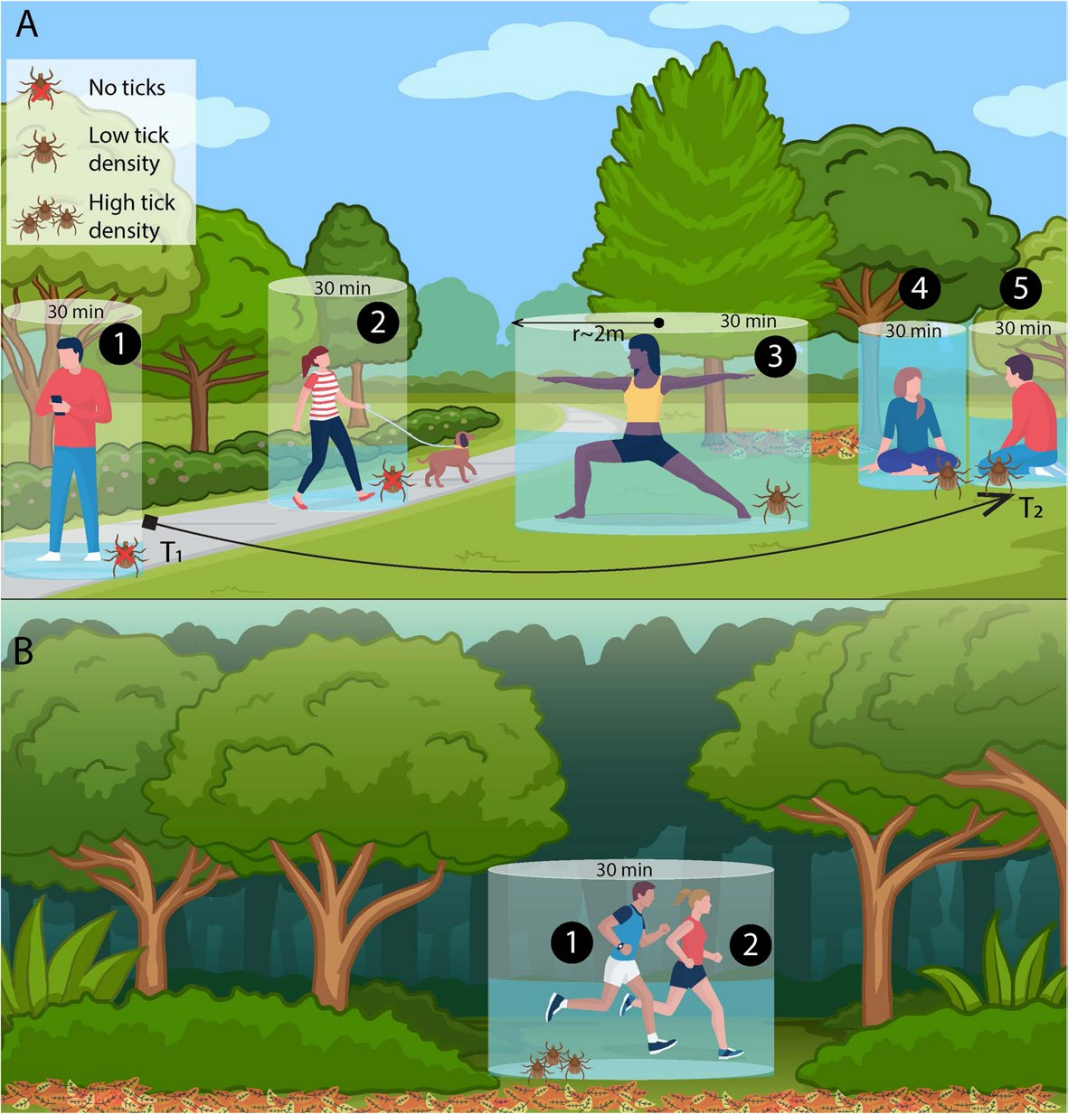
Examples of the proportion of time spent in a specific location within the park (**A** Open area; **B** Trail) that was integrated with the mean nymphal density per 10 m^2^ by habitat (maintained grass, unmaintained herbaceous, leaf litter, impervious surface), site (open space vs. trails), and park identity to estimate the probability of human-tick encounter. The cumulative probability of human-tick encounter was estimated for each data point or observation (the numbers in the figure) within the site. A new data point was assigned every time a person being observed moved to a different location within the area and the time elapsed in that focal point was recorded as well as the habitat type; thus, one person could have one observation during that time period or several depending on their movement (see T1 to T2 in panel A)

For open space sites, paper maps with landmark locations were used to record the specific location of participants within the site (i.e., data point or observation) (see Additional file 5). For each data point, we recorded the habitat used by the visitor and the time elapsed in minutes that the visitor spent in. A new data point was assigned every time a person being observed moved to a different location within the site (approximately more than 2 m from the previous focal point) during the 30 min period, so one person could have one observation during that time period or several depending on their movement (see T1 to T2 in Fig. 2A). If a visitor entered and exited the site in under 1 min, they were given an elapsed time of 0.1 minutes to reflect presence in the site. The following was recorded for each observed visitor: entrance/exit time of individual, dominant activity (e.g., walking, exercising, socializing, etc.), if they were with a dog, estimated age range in 10-year intervals, and observed gender. Ages were estimated in four categories: child (0-10), teen (10-20), adult (20-60), and senior (60+). For trails, entrance/exit time, age range, gender, and dominant activity was recorded. With this information, visitor counts (the number of unique visitors present during a specific observation session) by park, site type, and habitat were totaled and used for analysis. Furthermore, in open spaces where the activity of all individuals was visible, the mean number of minutes spent in each habitat for each age group and gender was calculated. During the process, observers did not engage with visitors to avoid influencing their behavior, and any individuals who approached observers were removed from the observational section of the study.

### Knowledge, attitudes, and practices survey

We administered a 10 min questionnaire to assess knowledge, attitudes, and practices regarding ticks and tick prevention (Additional file 6). The questionnaire was based on a previous KAP survey tool developed for vector-borne disease assessments on Staten Island using the health belief model framework [43]. The KAP survey was first implemented in the field in 2018 and was tested in focus groups with participants from the general population and adjusted accordingly based on the 2018 results and feedback from the focus group participants. It comprised 27 questions related to park use, knowledge of ticks and tick-borne diseases, attitudes about perceived risk and severity, tick prevention behavior, and demographics. Questions involved a mix of open-ended, close-ended ordered, and close-ended unordered responses. Demographic and background questions included age, gender identity, race/ethnicity, highest level of education received, park visitation frequency, activities engaged in at the park, and source of information for ticks and tick-borne diseases. Knowledge questions included tick identification (Additional file 7), tick habitat, tick exposure, acquisition of the Lyme disease bacterium, prevention methods, and tick removal. Attitude questions included perceived severity of tick-borne diseases on Staten Island, perceived likelihood of tick encounter, reasons for not checking for ticks, and concerns about repellent use. Practice questions included frequency of repellent use, personal protection measures against ticks, and frequency of tick checks.

Participants were recruited by convenience sampling when 30 min observations were concluded so as to not interfere with the observations. Individuals who were not actively engaged in an activity (e.g., talking on the phone, running, playing sports, etc.) were approached for the survey, and all refusals and refusal reasons were recorded. We explained the purpose of the study and interviewed individuals over 18-years-old who gave oral consent. Individuals were able to stop the survey at any time, and the responses of only one visitor, if in a group, were recorded. Prior to administering the questionnaire, our team was trained on how to approach participants and avoid selection bias, to deliver the questionnaire in a uniform way, and to avoid potential ways of biasing participant responses. Prior to its implementation, the questionnaire was piloted to improve delivery length. Survey administrators wore clothing with institutional logos and name tags to improve response rate.

Open-ended responses were categorized, and since the responses were non-mutually exclusive, the responses were turned into dummy variables (i.e., given a 0 or 1 if a given response was verbalized). Closed-ended |unordered questions were also turned into dummy variables. Closed-ended ordinal questions (perceived severity of ticks and tick-borne diseases and perceived likelihood of tick encounter) were recorded on a 5-point Likert scale.

### Data analysis

Analyses were performed using R 2019 (R Foundation for Statistical Computing, Vienna, Austria. URL: https://www.R-project.org/) with the following packages: ‘MASS’ [44], ‘emmeans’ [45], ‘MuMIn’ [46], ‘rms’ [47], and ‘car’ [48].

#### Tick density

We evaluated the association between tick counts per 100 m^2^ and park identity, site type (open spaces, edges of open spaces, trails), and habitat type (maintained grass, unmaintained herbaceous, leaf litter) as predictor variables. Our sampling strategy was timed to encompass the nymphal peak for all species, therefore the models only included unadjusted nymphal counts. We did not collect *I. scapularis* after week 10, so weeks 11 and 12 were removed from the analysis for this species. For the other tick species, data collected over 12 weeks was considered. Because tick counts were over-dispersed, a generalized linear model with a negative binomial error structure was selected for determining variables that best predicted tick counts for each species. The length of the transect was included as an offset in the model to account for differential effort. The models were performed separately for each tick species collected. Reference categories for park, site type, and habitat were selected based on the category with the most observations (number of drags) as the normative category to facilitate interpretation. For *I. scapularis* and *A. americanum* models, the park reference variable was Clove Lakes, the habitat reference was unmaintained herbaceous, and site type reference was trails. For *H. longicornis* models, habitat reference was unmaintained herbaceous and site type reference was trails; park was removed from the model since *H. longicornis* was only found in the Conference House location.

To account for model selection uncertainty, model averaging was implemented using the MuMin package by ordering competing models based on the Akaike’s information criterion (AIC) value [49]. If there was more than one model with a ∆AIC < 2 from the best ranked model, model averaging was performed [50]. Otherwise, the best ranked model was selected. Models were evaluated for multicollinearity issues by evaluating if the variance inflation factor (VIF) < 4, using the vif function from the rms package in R. If VIF > 4, we evaluated which variables were redundant, and decided on their inclusion based on our knowledge of the causal structure. The model coefficients were back-transformed from the log scale to determine the relative abundance of ticks per 100 m^2^ with respect to the reference category. The mean predicted tick number per 100 m^2^ (density of nymphs, DON) with confidence intervals was calculated for all model variables using emmeans: pairs and type = “response” in R.

#### Park visitor observations

Visitor counts and elapsed time were compared by park, site type, habitat, and habitat exposure time; they were examined by gender and across age groups. Counts were modeled using a generalized linear model with a Poisson error distribution with an interaction effect between age and habitat and gender and habitat. The significance of the interactions was determined with an Analysis of Deviance (type III) table and chi-square test. Interactions were analyzed using emmeans: pairwise and type = “response” with a Tukey method adjustment. To determine whether the mean elapsed time that visitors spent in different habitats differed by age group and gender, an additive linear regression model was used with the main effects of age group, gender, and habitat for each park. Subsequently, post-hoc pairwise comparisons between age groups and habitats were performed using emmeans: pairwise and type = “response” with a Tukey method adjustment.

#### Probability of human-tick encounter

We estimated the cumulative probability of a person encountering a nymph during a 30 min period as a measure of exposure risk per site type (open spaces vs. trails) and park (hereafter, probability of human-tick encounter). This measure was derived from the park visitor observations to assess the amount of time people spend in each specific location (or “focal point”) during the 30 min observation period and the mean nymphal density in that location (see Tick Density section). The probability of human-tick encounter was estimated for each focal point within each site assuming that the probability of human-tick encounter was independent among focal points, even for one individual recorded in multiple locations during the observation period. In other words, the probability of an individual encountering a tick in a focal point was independent of the same individual encountering a tick in another focal point. Therefore, the estimated probabilities of human-tick encounters can be considered a characteristic of the site type and the park, rather than a characteristic of the individuals. In each focal point, we estimated the proportion of time spent by any individual over the observed time period (30 min) and the mean nymphal density given the habitat, site type, and park (Fig. 2).

To estimate the probability of human-tick encounter, we used a passive sampling model that has been traditionally used to explain the species-area relationships [51]. This model assumes that the probability of finding a species in a defined area depends on the size of the “target” area and the number of randomly distributed individuals of said species (i.e. “darts”) [51]. Similarly, we can consider relative exposure time over an observation period (30 min) as the “target” and tick density as “darts”. We assumed the probability of any nymph missing a person in 30 min in a focal point was inverse to the fraction of the 30 min interval a person spent in the area (i.e., the longer the person stayed, the lower the probability they will ‘miss’ encountering a tick):

1 $$P(miss)=1-\frac{\Delta t}{P}$$

were *∆t* is the time elapsed in that focal point (in mins) and *P* is the total observational period (30 mins).

The probability of missing encountering all *n* ticks is thus:

2 $$P(miss)={\left(1-\frac{\Delta t}{P}\right)}^n$$

where *n* is the number of ticks in a defined area, and thus we can replace *n* (number of ticks) by *d* (density of ticks):

3 $$P(miss)={\left(1-\frac{\Delta t}{P}\right)}^d$$

Since we recorded a different data point when the person moved to a distinct location (more than 2 m) within the site, we used the estimated tick density per 10 m^2^ (~ 2 m radius around the focal point) determined by the habitat, site type and park (Fig. 2).

Finally, the probability of human-tick encounter for each observed focal point in a 30 min observation period can be estimated from the observed data as:

4 $$P(tick)=1-{\left(1-\frac{\Delta t}{P}\right)}^d\propto \int_{t0}^{t1-t0}e(t) dt$$

where e(t) is the probability of encountering a tick, which is bounded by the time spent in a specific location [29].

This probability was estimated in each focal point where a person was observed in the 14 sites were behavioral observations and tick drags were conducted, and comparisons were conducted between site types (open spaces vs. trails) and among parks.

For trails, since we were not able to record exit times in all cases, we simulated a negative binomial distribution for the time elapsed on trails using the variance from the observed data in open spaces (i.e., the variance in time elapsed per site), to obtain a more accurate measure of variability, and we used a median of 15 min (half of estimated period). We derived the time spent in a trail as random draws from this distribution.

The probability of human-tick encounter was estimated for each tick species individually. The code for the probability of tick encounters can be found at https://piliffq.github.io/tick-risk-index/.

#### Knowledge, attitudes, and preventative practices of park visitors

In this study, we focused on predictors of performing tick checks among park users given that tick checks can be conducted by nearly every member of the public, irrespective of access or attitudes towards personal protective equipment, clothing, and repellent. CDC guidelines indicate tick checks are one of the most effective ways to prevent tick attachment and the transmission of tick-borne pathogens, making it the single most important tick-borne disease preventative method to practice. The KAP survey questions were organized into five sections: demographics, prior experience, knowledge, attitudes, and practices. Race/ethnicity was converted into a binomial variable (white or other) given most respondents identified as white. Education responses were grouped into “High school or less”, “Some college/Associate”, “Bachelors”, and “Graduate”. Age was categorized into six groups: 18-28, 29-39, 40-50, 51-61, 62-72, and 73-83. Questions related to prior tick experience were grouped and given a score from 0 to 4 (if yes to all, score was 4). This included whether the respondent had seen a tick before, found a tick on a pet or household member, and whether someone in the home had been diagnosed with Lyme disease. Knowledge questions were scored based on the number of correct responses, and individuals received one point per correct response. Identification knowledge score was out of sixteen points, and individuals received one point for every specimen they correctly determined was a tick and one point for every non-tick they identified correctly. Respondents received a tick habitat and bacterium acquisition score for every correct habitat they identified where ticks could be found and every correct response for how ticks can become infected with the Lyme disease bacterium. They also received a score for the total number of correct tick prevention methods they could identify, with one point for each correct method. Questions regarding knowledge and practices for tick prevention methods were open-ended. Some individuals reported practicing certain prevention methods in the practices portion of the survey but failed to report knowing about these methods in the knowledge section. In these cases, individual knowledge scores were categorized to reflect practicing the behavior. Attitude questions involving perceived severity of tick-transmitted diseases on Staten Island and perceived likelihood of tick encounter were ordered on a Likert scale and scored out of five, with five being the most severe or most likely.

Generalized linear models were used to determine which variables influenced previous tick exposure, perceived severity of tick-borne diseases, and perceived probability of tick encounter. Explanatory variables included in each model (excluding the variable being modeled) were park, park visitation frequency, perceived severity of tick-borne diseases, perceived probability of tick encounter, number of prevention methods used, knowledge scores for tick identification, habitat, and prevention methods, owning a dog, education level, gender, and age group. Linear models were performed to assess factors influencing the knowledge scores for tick identification, habitat, and prevention methods with the same explanatory variables included as described above. Emmeans was used for post-hoc comparisons for categorical variables, and *p*-values were adjusted using the Tukey method. Tick check frequency was converted into a binomial variable (“yes” to always or sometimes checking for ticks and “no” for never checking for ticks) and analyzed using a generalized linear model with a binomial regression. Model selection was conducted as described above.

## Results

### Tick hazard in parks

From May to August 2019, 432 drags were performed (Clove Lakes: *n* = 168; Conference House: *n* = 134; Willowbrook: *n* = 132) with each park visited once per week when weather allowed. All three habitats (maintained grass, leaf litter, and unmaintained herbaceous) were present in Clove Lakes and Willowbrook; however, no leaf litter habitat was present in Conference House (Additional file 3).

A total of 10,036 ticks were collected across all parks and sites, including 7133 *H. longicornis* (adults: *n* = 489; nymphs: *n* = 2599; larvae: *n* = 4045), 1972 *A. americanum* (adults: *n* = 28; nymph: *n* = 157; larvae: *n* = 1787) and 931 *I. scapularis* (adults: *n* = 0; nymphs: *n* = 85; larvae: *n* = 846) (Additional file 8), with temporal variation by life stage (Additional file 9). We focused on the nymphal stage that is associated with the highest risk for the transmission of tick-borne pathogens to humans.

Model results showed that the densities of *I. scapularis* and *A. americanum* nymphs were best explained by park and habitat, with the greatest densities in Conference House, and the lowest densities in maintained grass (Table 1). The density of *A. americanum* also was explained by site type: the density of nymphs at the edge of open spaces was lower compared to trails; no difference was observed between the latter and the density found in vegetated open spaces (Table 1, Additional file 10). Lastly, *H. longicornis* nymphs were only found in Conference House, and the density of this species was best described by site type and habitat (Table 1). Similar to the other tick species, density of *H. longicornis* nymphs was lowest at the edge of open spaces and in maintained grass (Additional file 10).

**Table 1:** Generalized linear model (negative binomial regression) summary for each species. Drags were performed within open spaces or at the edge of open spaces (“Edge”). Relative abundance (RA) is the expected log count of nymphs for each unit of increase of the categorical variable compared to the reference variable, or incidence rate ratio (IRR). The mean density of nymphs (DON) refers to the model predicted number of nymphs per 100 m^2^. For *A. americanum* the best model with the lowest AIC is presented, and no competing models were within 2 AIC. Model averaging was conducted for *H. longicornis* and *I. scapularis. H. longicornis* was only found in one park (Conference House), and no leaf litter habitat was present in this park among the areas frequently visited

Variable	Category	RA;	***p***-value	Mean DON;
		95% CI		95% CI
***I. scapularis***				
**Intercept**		0.002 (0, 0.014)	< 0.001	
**Park**	Clove Lakes	1		7.96 (0.98, 64.7)
	Conference House	4.43 (1.72, 10.83)	0.006	33.09 (4.04, 270.9)
	Willowbrook	0.89 (0.34, 2.32)	0.806	7.06 (0.87, 57.4)
**Site type**	Trails	1		12.5 (1.67, 9.41)
	Open space	1.15 (0.35, 3.84)	0.927	12.8 (1.69, 97.3)
	Edge	0.62 (0.20, 1.88)	0.790	11.6 (1.45, 92.6)
**Habitat**	Unmaintained herbaceous	1		31.71 (4.58, 219.7)
	Maintained grass	0.04 (0.01, 0.34)	0.003	1.39 (0.09, 22.2)
	Leaf litter	1.33 (0.48, 3.71)	0.583	42.25 (5.46, 326.9)
***A. americanum***				
**Intercept**		0.0004 (0, 0.001)	< 0.001	
**Park**	Clove Lakes	1		1.13 (0.33, 3.83)
	Conference House	33.09 (8.12, 134.95)	< 0.001	37.44 (17.93, 78.21)
	Willowbrook	1.69 (0.36, 7.83)	0.502	1.91 (0.66, 5.52)
**Site type**	Trail	1		7.51 (3.38, 16.68)
	Open space	0.87 (0.23, 3.36)	0.840	6.54 (2.49, 17.18)
	Edge	0.22 (0.07, 0.72)	0.012	1.65 (0.44, 6.22)
**Habitat**	Unmaintained herbaceous	1		8.97 (3.94, 20.42)
	Maintained grass	0.17 (0.04, 0.78)	0.023	1.50 (0.38, 5.96)
	Leaf litter	0.67 (0.14, 3.13)	0.611	6.01 (1.67, 21.68)
***H. longicornis***				
**Intercept**		0.24 (0.17, 0.32)	< 0.001	
**Site type**	Trails	1		762 (327.1, 1777)
	Open space	0.43 (0.09, 1.84)	0.258	380 (174.2, 827)
	Edge	0.26 (0.11, 0.63)	0.003	254 (72.8, 885)
**Habitat**	Unmaintained herbaceous	1		1435 (689.1, 2990)
	Maintained grass	0.09 (0.02, 0.38)	0.001	122 (41.7, 358)

### Outdoor activity patterns by park visitors

During the study period, a total of 5910 individuals were observed entering the parks over 294 observation hours. Across all parks, 3214 visitors were men and 2632 were women (*P* < 0.001), and more adults visited the parks compared to children, teens, and seniors (*P <* 0.0001 for all comparisons) (Table 2). Most people visited Clove Lakes (*n* = 2773), followed by Willowbrook (*n* = 1975) and Conference House (*n* = 1162) (*P <* 0.001 for all comparisons). Gender distribution among park visitors was similar (Table 2).

**Table 2:** Counts of park visitors by age group and gender. The total number (n) and percent of total visitors (%) in each park within each group

Park	Gender n (%)		Age group n (%)			
	Male	Female	Child	Teen	Adult	Senior
Clove Lakes	1583 (56.8)	1194 (43.2)	200 (7.2)	207 (7.4)	1877 (67.7)	490 (17.7)
Willowbrook	1032 (52.4)	945 (47.6)	402 (20.3)	174 (8.8)	1187 (60)	214 (10.8)
Conference House	649 (55)	529 (44.9)	288 (24.3)	151 (12.7)	582 (49)	166 (14)

Generally, open spaces were used more than trails, and impervious surfaces were used more than habitats with vegetation, but visitor counts in each site type and habitat varied by gender and age group within each park in different ways (see Additional file 12). Gender distribution was similar in trails and open spaces in Clove Lakes and Willowbrook (*P* = 0.0958 and *P* = 0.708), but in Conference House, males visited trails more compared to females (*P* < 0.0001). Children used open spaces more often than older age groups in Willowbrook (*P* < 0.0001) and Conference House (*P* < 0.0001) but not in Clove Lakes (*P* = 0.6794). Regarding habitat use, differences in gender distribution among habitats were also observed in Willowbrook and Conference House, with males visiting unmaintained vegetation or leaf litter habitats more than females (*P* = 0.0027 and *P* < 0.0001, respectively), but no significant differences were observed in Clove Lakes (*P* = 0.056, respectively).

Across all parks, visitors were exposed to maintained grass habitats for the longest time duration (Additional file 13). In Clove Lakes, exposure time to different habitats was also influenced by age but not gender (*P* < 0.0001 and *P* = 0.2927, respectively) with children staying in the grass longer than teens, adults, and seniors (*P* < 0.0001 for all comparisons). In Conference House, exposure time varied by age and gender (*P* = 0.0049 and *P* = 0.002, respectively) with children exposed longer than teens and adults (*P* = 0.0258 and *P =* 0.0049, respectively) and females exposed longer than men (*P* = 0.002). Age and gender did not impact habitat exposure times in Willowbrook (*P* = 0.2930 and *P* = 0.9723, respectively). Picnicking (range of average duration times recorded across all parks: 16-25 min), socializing (13-28 min for all parks), exercising (2-29 min), sitting (13-14 min), tanning (8-20 min), and engaging in arts and photography (4-25 min) were long-stay activities that occurred in maintained grass habitats, potentially exposing many individuals to low levels of ticks, especially if occurring near a forested edge with leaf litter. The most frequent activities recorded in more hazardous habitats (leaf litter and unmaintained herbaceous) were short-stay activities, such as walking (range of average duration across all parks:< 1-3 min), jogging (< 1 min for all parks), biking (< 1-1 min), and working (3 mins for all parks) (Additional file 14).

### The probability of human-tick encounters in parks

**Fig. 3:**
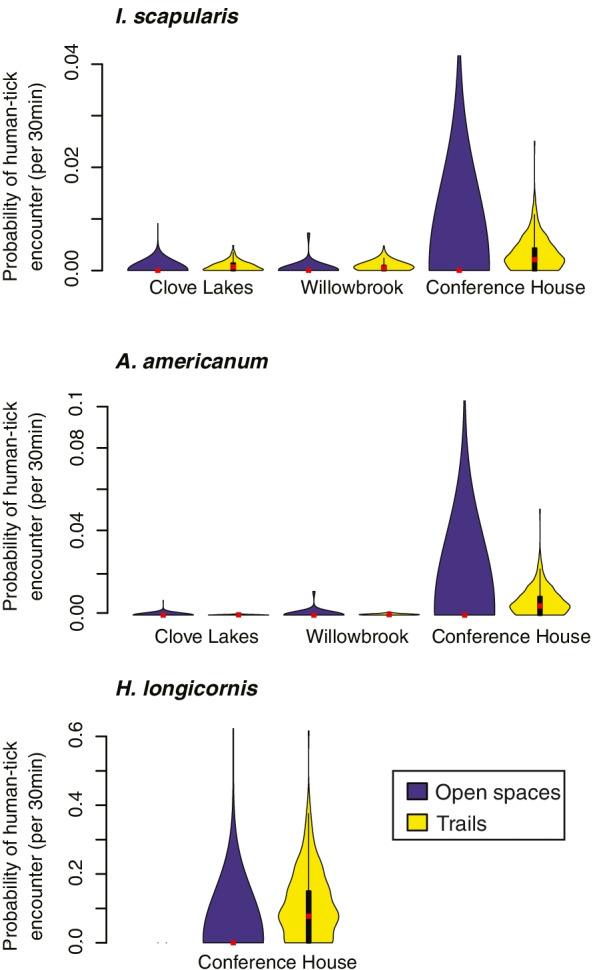
Distribution of the probabilities of human-tick encounter across park and site type, given the time spent by park visitors in each site and the mean nymphal densities per species

When combining the tick density estimated at each location (tick hazard) with the human exposure time estimated over a period of 30 min through observations, we found that the probability of human-tick encounter was higher in trails compared to open areas for all species (see Fig. 3). During a 30 min period, park visitors had a small probability (median = 0.1%; IQR: 0-0.2%) of encountering an *I. scapularis* nymph when transiting trails, but the probability of encountering an *I. scapularis* nymph in open areas was almost zero (median probability: 0% IQR: 0-0%, Q_99_: 0.7%). A similar pattern was observed for *A. americanum*, with a median 0.02% (IQR: 0-0.08%) probability of finding a nymph in trails and an almost zero probability of encountering them in open areas (median probability: 0% IQR: 0-0%, Q_99_: 1.4%). For both species, the highest probability of encountering a nymph was estimated for Conference House (Fig. 3). Lastly, *H. longicornis* was only found at Conference House, and the probability of finding a nymph was also higher in trails compared to open areas (median probability: 7.7%, IQR: 0-15.0% in trails and median probability: 0%, IQR: 0-0%, Q_99_: 40.3% in open spaces). Although the probability of finding a tick in open areas was almost zero for all species, the ranges of estimated human-tick encounter probabilities were highly variable, and non-zero probabilities were estimated for all open spaces (Fig. 3).

### Prior experiences with ticks and tick-borne diseases among park visitors

In total, 190 KAP surveys were administered across all parks (Clove Lakes: *n* = 65; Willowbrook: *n* = 61, Conference House: *n* = 64). The refusal rate to participate in the survey was 18% (52% were male, and 46% were 50-70 years old). Participants were predominately residents of Staten Island (*n* = 176; 93%) with a median age of 50 years (range:18-82), and more frequently male (*n* = 109; 57.4%) and non-Hispanic/Latinx white/Caucasian (*n* = 112; 58.9%). Most attended some college or received a 2-year college (associates) degree (*n* = 71; 37.4%) (Additional file 15).

While most visitors attended the parks regularly, few and inconsistent personal protective measures were used to limit tick exposure (Additional file 15). Thirty-six percent (*n* = 69) of respondents reported visiting parks daily, 18.4% (*n* = 35) several times a week, 10% (n = 19) once a week, 10.5% (*n* = 20) once a month, 16.3% (*n* = 31) once a year, and 6.8% (n = 13) visited for the park for the first time. Walking/running (*n* = 102; 53.7%), dog walking (*n* = 39; 20.5%), and relaxing (n = 28; 14.7%) were the top activities enjoyed by respondents.

Regarding their past experience with ticks and tick-borne diseases, 60% of visitors (*n* = 114) reported seeing a tick before. Of those who had seen a tick, 53.5% (n = 61) reported finding ticks on either themselves or a household member, and 54.4% (n = 61) had found ticks on a pet. Forty-four percent of respondents (n = 84) knew someone with Lyme disease, and 18% (n = 19) reported a past Lyme disease diagnosis of a household member. However, prior experience with ticks did not vary among parks and was not associated to park visitation frequency, knowledge of tick habitat, knowledge of tick species and stage, perceived probability of tick encounter, knowledge of tick prevention methods, number of prevention methods used, perceived severity, gender, age, education, or owning a dog (*P >* 0.05).

### Knowledge and attitudes regarding ticks and tick-borne diseases

The two main sources for acquiring information about ticks and tick-borne diseases were the internet (*n* = 77; 41.4%) and TV/radio (*n* = 37; 19.9%). Most park visitors were able to distinguish at least one tick specimen from other arthropods in a sample of eight arthropods (Additional file 6), but when asked if they knew which specimens were ticks, only 38.1% (*n* = 43) and 26.5% (*n* = 30) of participants were able to recognize *A. americanum* adults or *I. scapularis* adults, respectively. Only 3.5% (n = 4) of participants were able to recognize *I. scapularis* nymphs as a tick. Regarding tick habitat, most individuals identified parks in general as the main source for tick exposure (*n* = 82; 43.2%), followed by more specific habitat identification such as woods (*n* = 52; 27.4%) and grass (*n* = 32; 16.8%) (Additional file 15).

Regarding knowledge of tick prevention measures, 17.4% (*n* = 33) of individuals did not know any prevention measures, and of the respondents who were aware of prevention methods, they mentioned a median of two measures (Additional file 15). Of all respondents, 62% (*n* = 117) knew about insect repellent, 40% (*n* = 76) about wearing long sleeves, 24.7% (*n* = 47) were familiar with tick checks, and 23.2% (*n* = 44) reported knowing about tucking pants into socks or wearing long socks. Fewer people reported knowing about wearing light colored clothing to spot ticks easily (n = 15; 7.9%) and showering after being outdoors (n = 10; 5.3%). In addition, 35.8% (*n* = 68) mentioned avoiding tick habitat as a preventative measure. When asked about how Lyme disease cases can be reduced on Staten Island, most believed that spraying pesticides (*n* = 63; 34.9%), generally educating the public (*n* = 55; 29.6%), and reducing or controlling deer (n = 33; 17.7%) would control the disease, but fewer people reported the use of personal protection measures (n = 15; 8.1%) as an effective way of reducing cases on Staten Island.

Perceptions of tick-borne diseases as a public health problem on Staten Island among participants was highly variable: 42.1% (*n* = 80) of participants considered Lyme disease as either an extremely serious or very serious problem, 15.8% (n = 30) believed it was not at all serious or slightly serious, and 20% were unsure about the status of tick-borne diseases on Staten Island (*n* = 38). People with a higher perception of severity had higher perceived probability of tick encounter (*P =* 0.01). However, knowledge about preventative measures as well as perceived level of severity of tick-borne diseases on Staten Island, did not vary by park, and was not associated with visitation frequency, prior experience with ticks, education, gender, age, or owning a dog (*P >* 0.05 for all comparisons).

### Preventative practices against ticks and tick-borne diseases

Although more than 80% of respondents knew at least one preventative measure, 32.8% (*n* = 62) did not practice any preventative measures. On the other hand, 30.7% (*n* = 58) of respondents practiced one, 19% (*n* = 36) practiced two, 10.6% (n = 20) practiced three, and 6.9% (n = 13) practiced four or more. The prevention methods most frequently mentioned were avoiding tick habitat (n = 58; 30.7%), using repellent (*n* = 54; 28.5%), wearing long sleeves (*n* = 49; 25.9%), and conducting tick checks (*n* = 34; 18%). Fewer people reported tucking pants into socks or wearing long socks (*n* = 26; 13.8%), wearing light colored clothing (n = 7; 3.7%), or showering after being outdoors (n = 8; 4%).

When specifically asked about checking for ticks after being outdoors, more than half of the respondents (*n* = 99; 58%) reported conducting tick checks either sometimes or always, while 42% (*n* = 79) reported never checking for ticks (Additional file 15). Those who never checked for ticks believed they were not in an area with ticks (24%; *n* = 46), were too lazy (12%; *n* = 23), forgot to check (12%, *n* = 22), never had experience with ticks (10%, n = 19), or did not think about it at the time (10%; n = 19). Results from the generalized linear model (Table 3) showed that tick check behavior could be best predicted by the number of tick prevention methods known, the perceived probability of tick encounter, and knowledge of tick habitat. The odds for conducting tick checks increased almost two-fold with the number of prevention methods they knew. Likewise, the odds of checking for ticks increased 1.6 times with each unit increase in the perceived probability of encountering a tick. The odds of a park visitor checking for ticks who perceived their risk for tick encounter as “extremely likely” was 6.9 times higher than a person who perceived their risk for tick encounter as “very unlikely”. Accurate knowledge about tick habitat was not associated with performing tick checks.

**Table 3:** Generalized linear regression model for predicting tick checks. The odds of checking for ticks were obtained by exponentiating the estimate

Predictor	Coefficient	SE	*p*-value	Odds ratio
Intercept	−0.7419	0.5881	0.2712	
Number of prevention methods known	0.6605	0.2235	0.00312	1.9
Perceived probability of tick encounter	0.4845	0.1711	0.00464	1.6
Knowledge of tick habitat	−0.6434	0.3508	0.06664	0.5

## Discussion

To the best of our knowledge, this is the first study to integrate the tick hazard with simultaneous assessment of human behaviors linked to the risk of tick exposure [3]. Previous studies have examined risk for tick encounter based on acarological indices [15, 16]; however, risk also depends on human usage of parks (selected areas for recreation, type of activity, and time spent) and protective behaviors to prevent tick bites and the transmission of tick-borne pathogens. Furthermore, we showed that people’s KAP did not change across parks even if parks represented different exposure risks.

Consistent with previous studies reporting heterogenous tick densities across parks [16–19, 31], Conference House, at the southern tip of Staten Island, had the highest density of ticks of all species. However, the fewest individuals visited this park, and the main tick present was *H. longicornis*, which does not frequently bite humans or transmit known pathogens to humans in the United States [52–54]. This tick is a new introduction to Staten Island and may become more abundant in other Staten Island parks in the future. While the high density of these ticks may not be cause for pathogen concern at this moment, it is still possible that the presence of these ticks may affect human behavior. For example, the high density of *H. longicornis* or the more aggressive host-seeking behavior of *A. americanum* ticks that increase tick-human/pet encounters may act as cues to implement preventative behaviors against ticks, impacting the risk of encountering more passive *I. scapularis* ticks and reducing disease transmission to humans. Moreover, *I. scapularis* co-occurred with the other tick species, given the wider range of ecological niches that the other two tick species commonly exploit.

Trails, unmaintained herbaceous, and leaf litter habitats were the most hazardous areas, but fewer people chose to use these habitats, opting to use impervious surfaces and maintained open grass habitats, limiting their risk for tick exposure. The individuals choosing to visit hazardous habitats (unmaintained herbaceous and leaf litter) and site types (trails) were most often males and adults. Furthermore, different types of activities may put park visitors at risk if the activities are performed in hazardous habitats and if they expose individuals to hazardous habitats for longer periods of time; however, the activities performed in these areas were short-stay activities. Moreover, some people, mainly children and females at Conference House, spent the most time in maintained grass, and, while tick densities were low in this habitat, there was still a minimal risk for tick exposure, primarily exposure to *H. longicornis* which can be encountered in maintained open grass habitats.

This mismatch between the tick hazard and human exposure to the hazard was reflected in the estimated probabilities of human-tick encounter, which can be interpreted as the baseline risk of encountering a tick by a person in a set time interval (the observation period). It is important to observe that the probability of tick encounter derived here simply formalizes the association between tick density and time spent, assuming simple passive sampling (i.e, the accumulation of ticks based on the time spent and the number of ticks in a focal point). This baseline risk can, of course, be modified by preventative practices that people might use, so our estimations assume the simplest case in which no preventative practices are undertaken to prevent tick exposure. Moreover, this tick encounter probability does not aim to capture the individual risk per person (which would be modified by preventative practices and the total number of sites visited where tick exposure can occur) but estimate the human-tick probability per park location by going a step beyond acarological indices and integrating human exposure patterns. The estimated probability of human-tick encounter in Staten Island parks shows that the risk of tick-encounter was higher in trails compared to open spaces. However, although the estimated probability of tick encounter in a 30 min period spent in open spaces was almost zero, the probability of tick-encounter was highly variable, which was mostly driven by the variability of time spent in an area and the specific location of the activity. Together, these factors can create highly heterogeneous tick exposure among park visitors.

Managing Lyme and other tick-borne disease risks in peridomestic settings and natural areas differ in the fact that while humans can reduce the density of ticks by conducting environmental interventions in their yards (e.g., applying area-wide acaricides, treating rodents for ticks, or by performing landscape modifications) [6, 55], these interventions are not feasible at larger scales or on public land. Thus, recreational park visitors can only manage their risk by adjusting their behavior to reduce the chance of tick exposure or minimize the risk of transmission by promptly removing attached ticks. Commonly advised protective behaviors against tick-borne diseases include avoiding high-risk areas by staying in the center of trails, applying tick-repellent, wearing protective clothing (i.e., permethrin-treated pants), and checking for ticks and/or showering after spending time in risky environments [56].

Our survey results indicated that respondents believed they personally had little risk for tick encounter, even though many park visitors thought tick-borne diseases were serious and exposure to ticks was mainly from parks. This perception could have influenced their tick bite risk. The likelihood of practicing tick checks was increased by visitors knowing multiple prevention methods and their perceived probability of tick encounters. Similarly, Donohoe et al. (2018) found that tick checks were influenced by having more knowledge of tick prevention and perceiving a higher risk for tick-borne disease exposure [57]. However, Butler et al. (2016) found that practicing tick checks was related to self-reported history of disease, and those previously infected with a tick-borne disease were more likely to perform tick checks [58]. In our study, prior experience with ticks or tick-borne disease was not associated with increased knowledge of preventative practices. Checking the body for ticks after being outdoors (and removing ticks if found) remains one of the best ways to decrease the chances of acquiring a tick-borne infection, and public health education about ticks in parks would increase awareness and should encourage practicing multiple tick preventative behaviors, highlighting frequent tick checks when outdoors.

### Limitations

Regarding tick sampling, variability with the GPS tracking apps provided fluctuating levels of accuracy when tracking our dragging locations. In tracking human usage of park spaces, we did not account for unique visitor counts since it was not possible to know if the same individuals returned to the same park on different days. Thus, we were not able to adjust for heterogeneities in individual behaviors as the same individuals might have returned to the park multiple times across the summer. We could also not verify the length of time that visitors spent in trails or the frequency of contact between visitors and trailside vegetation (where ticks were sampled) since we were located at the head of the trail, and we only recorded entry and exit times. Moreover, we estimated age and gender of park visitors when conducting the observations visually, thus, it is possible to have mistakenly misgendered park visitors as well as misclassified their age category (child, teen, adult, senior), limiting the demographic analyses of park use data. When administering the KAP surveys, we could not interview individuals who did not speak English, and we could only interview people who were not actively engaged in an activity. Therefore, we were not able to match individual behaviors and risk of tick encounters to the knowledge, attitudes, and practice surveys, and comparisons were done qualitatively at the park level. Moreover, children and teens went into hazardous locations in the parks; however, we could not interview visitors under 18 years of age.

Lastly, the cumulative probability of human-tick encounter estimated from the data is bounded by the observational period (0-30 min). Thus, if a person stayed during the whole observation period in the same location (at the upper limit), then *P(tick) = 1,* meaning that the cumulative probability of finding a tick is saturated and becomes independent of tick density. In theory, this would be correct if we consider the limit to infinity (a sufficiently large time period *P*). However, it is uncertain whether a tick would find a person within a 30 min period at a focal point even if present within 2 m. We avoided this issue at the limit by setting the proportion of time elapsed to 0.96 (29 min) instead of 1 if the time elapsed at a given location was 30 min. This risk index was intended to be a proof of concept to integrate human behavioral information and tick density based on previous human-vector encounter models. Future variations of this probability could expand to validate the appropriate observation periods, include host preference by the different tick species and questing activity thought the day.

## Conclusions

Our results from three Staten Island, NY, parks provide the first simultaneous information on tick bite risk and human behavior. We identified high variability in tick exposure risk across these parks within the same urban area, highlighting the importance of understanding local park usage and human behavior in conjunction with tick density assessments at distinct locations to adequately calculate risk while understanding visitors’ motives for tick prevention. Future studies that aim to understand visitor behaviors and preferences alongside real-time risk assessments could enable a refined understanding of habitat and tick control measures. In addition, these approaches could lead to more impactful park visitor education practices, ultimately contributing to a greater overall sense of well-being and visitor confidence in local parks and the multitude of green space benefits to personal and public well-being.

## Supplementary Information

**Additional file 1.** Description of the 14 sites used in this study.

**Additional file 2.** Description of habitats classified in the park sites.

**Additional file 3.** The number of drags (n) conducted in each site type (light gray) within each park. The drag habitats found across the parks were A) maintained grass, B) leaf litter, and C) unmaintained herbaceous. At the edge of open spaces, the drag habitat comprised of the habitat where the drag was performed, but the habitat on either side of the dragged habitat was also recorded to characterize edge type. Edge drag habitats could be found in between 1) impervious and forest, 2) maintained grass and forest, or 3) maintained grass and water environments.

**Additional file 4.** List of plant species found in unmaintained herbaceous and leaf litter habitats.

**Additional file 5.** Example map of park visitor movement within an open space. Human movement was mapped using arrows to denote the directional movement of each individual. Elapsed time was noted when visitors remained in one location. All daily time intervals for one site are represented: red = 9 am-12 pm, blue = 12 pm-3 pm, yellow = 3 pm-6 pm.

**Additional file 6.** KAP survey.

**Additional file 7.** Arthropod samples including ticks and non-tick specimens. Respondents were asked to distinguish which were ticks given 1) Eastern ash bark beetle, 2) American dog tick adult, 3) swallow bug, 4) drugstore bug, 5) lone star tick adult, 6) deer tick adult, 7) flea, and 8) deer tick nymph.

**Additional file 8.** Counts (n) and density (d) per 100 m2 of tick larvae (L), nymphs (N), and adults (A) in each park and site.

**Additional file 9. **Tick phenology in Staten Island parks. The proportional activity of each life stage from late- May to mid-August for *A. americanum*, *I. scapularis* and *H. longicornis* are shown.

**Additional file 10.** Nymph counts (n) and density per 100 m2 (d) by site type and park. Open spaces had either an edge (E) or no edge (NE).

**Additional file 11.** Nymph counts (n) and density per 100 m2 (d) by drag habitat and park.

**Additional file 12.** Counts of park visitors by age group and gender in each site type and habitat. The total number of unique visitors (n) and within-group percentage (%) of visitors in each site and habitat. Habitats include impervious (I), maintained grass (MG), leaf litter (LL), and unmaintained herbaceous (UH), with LL and UH being the most hazardous for tick encounter. NA denotes that the habitat type was not present in the park. Hyphenated habitats indicate that a visitor passed through two different habitats in a single movement event. Visitors may be recorded multiple times if they spent time in multiple habitats during their visit.

**Additional file 13.** The counts of visitors (n) and average time spent (minutes) in each habitat by age group and gender. Habitats include impervious (I), leaf litter (LL), maintained grass (MG), and unmaintained herbaceous (UH). Only data from open spaces is represented since elapsed time on trails could not be captured, and NA denotes that the habitat type was not present in the park open spaces. Hyphenated habitats indicate that a visitor passed through two different habitats in a single movement event. Visitors may be recorded multiple times if they spent time in multiple habitats during their visit.

**Additional file 14.** Counts of visitors (n) engaging in an activity and average elapsed time (min) spent in each park habitat. Information includes only includes open space site types because time spent could not be captured for individuals in trails. Individuals may be recorded multiple times if their activities and habitat usage changed during their visit in the site.

**Additional file 15.** KAP responses. Asterisk denotes multiple responses allowed.

## Data Availability

The datasets used and/or analyzed during the current study are available from the corresponding author on reasonable request.

## Abbreviation

KAP: Knowledge, Attitudes, and Practices
