# Supplementary material for: Integrating tick density and park visitor behaviors to assess the risk of tick exposure in urban parks on Staten Island, New York
Source: BMC Public Health. 2022 Aug 23;22:1602. doi: 10.1186/s12889-022-13989-x (PMC9396585; doi:10.1186/s12889-022-13989-x)
Supplement: Supplementary file 1 — Additional file 1. Description of the 14 sites used in this study. [file 12889_2022_13989_MOESM1_ESM.pdf]

Additional File 1. Description of the 14 sites used in this study.

Park	Site	Coordinates	Description
**Clove Lakes**	Open Space 1	*40°37'27.9"N 74°07'10.3" W*	Contains one paved pathway extending through the site with a large mowed green space that is bordered by a lake on one side. The opposite side is a wood line. There are two locations for exercise.
	Open Space 2	*40°37'22.8"N 74°07'02.0" W*	Includes one paved pathway that branches to lake access. Habitat is mainly mowed lawn space but is bordered by a wooded hillside with leaf litter.
	Open Space 3	*40°37'04.0"N 74°06'39.1" W*	Contains 14 picnic tables in an irregularly mowed grassy area. Two green spaces are dissected with a paved pathway, and the whole space is surrounded by woods. A natural trail borders one side.
	Trail 1	*40°37'12.0"N 74°06'49.9" W*	Natural trail characterized by shade and leaf litter. Entrance is near the Clove Lake.
	Trail 2	*40°37'05.5"N 74°06'47.0" W*	Natural trail characterized by shade and leaf litter. Entrance is near Open Space 3.
	Trail 3	*40°37'08.2"N 74°06'31.1" W*	Natural trail running parallel to paved trail at the main park entrance.
**Conference House**	Open Space 1	*40°30'10.8"N 74°15'13.0" W*	Characterized by a large mowed lawn, a community garden, two natural trails, and beach access. The historical Conference House is located here and attracts many visitors for recreational activities
	Open Space 2	*40°29'57.0"N 74°14'41.6" W*	Characterized by the Lenape Playground. Contained in this space is a mixture of mowed grass, a tall grass border, and large impenetrable ground space for the playground.
	Trail 1	*40°30'07.4"N 74°15'06.4" W*	Natural trail lined by forest and tall grass. Trail head begins at visitor center and runs along beach, the sand dunes, wetland, and meadow.
	Trail 2	*40°29'54.6"N 74°14'46.5" W*	Trail head located near the playground and wetland; characterized by tall grass with beach access.

**Willowbrook**	Open Space 1	*40°36'25.3"N 74°09'26.7" W*	Near park entrance and characterized by mowed lawn space, multiple paved pathway entrances, a lake, and one natural trail.
	Open Space 2	*40°36'11.0"N 74°09'30.0" W*	A large, shaded, mowed grassy space with 24 picnic tables, a woodline, natural trail access, and a Carousel attraction.
	Trail 1	*40°36'18.8"N 74°09'27.8" W*	Natural wooded trail that runs alongside the lake, connecting Open Space 1 to the visitor center.
	Trail 2	*40°36'06.7"N 74°09'26.2" W*	Forested trail located on the edge of Open Space 2; 7.6 miles in length and characterized by leaf litter and shade
