# Supplementary material for: Integrating tick density and park visitor behaviors to assess the risk of tick exposure in urban parks on Staten Island, New York
Source: BMC Public Health. 2022 Aug 23;22:1602. doi: 10.1186/s12889-022-13989-x (PMC9396585; doi:10.1186/s12889-022-13989-x)
Supplement: Supplementary file 2 — Additional file 2. Description of habitats classified in the park sites. [file 12889_2022_13989_MOESM2_ESM.pdf]

Additional File 2. Description of habitats classified in the park sites.

Habitat	Description
**Impervious**	Packed earth stripped of vegetation from overuse (bare earth) and/or environments void of plant material due to paved and other man-made, built environments
**Ecotone edge**	Two habitats converging (e.g. forest alongside maintained grass)
**Leaf litter**	Layer of leaf material, typically found under tree canopy
**Maintained grass**	Non-woody graminoids generally characterizing mowed lawn space
**Unmaintained herbaceous**	Non-woody herbaceous plants, a mixture of short weeds under 3 ft and over 3 ft
