# Supplementary material for: Integrating tick density and park visitor behaviors to assess the risk of tick exposure in urban parks on Staten Island, New York
Source: BMC Public Health. 2022 Aug 23;22:1602. doi: 10.1186/s12889-022-13989-x (PMC9396585; doi:10.1186/s12889-022-13989-x)

Additional File 3. The number of drags (n) conducted in each site type (light gray) within each park. The drag habitats found across the parks were A) maintained grass, B) leaf litter, and C) unmaintained herbaceous. At the edge of open spaces, the drag habitat comprised of the habitat where the drag was performed, but the habitat on either side of the dragged habitat was also recorded to characterize edge type. Edge drag habitats could be found in between 1) impervious and forest, 2) maintained grass and forest, or 3) maintained grass and water environments.

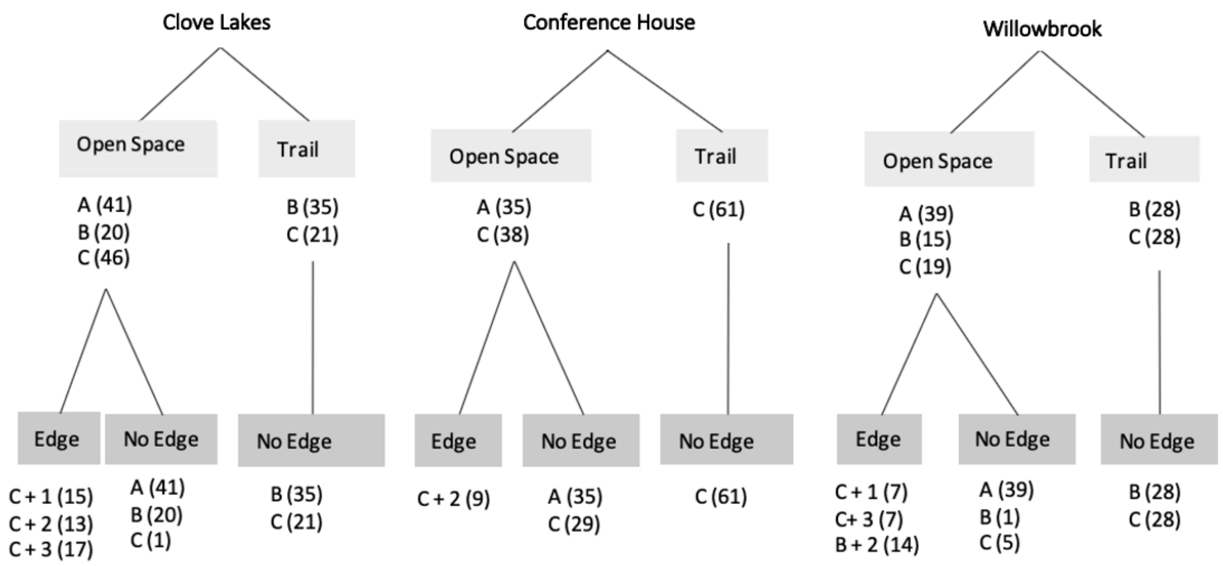

Supplement: Supplementary file 3 — Additional file 3. The number of drags (n) conducted in each site type (light gray) within each park. The drag habitats found across the parks were A) maintained grass, B) leaf litter, and C) unmaintained herbaceous. At the edge of open spaces, the drag habitat comprised of the habitat where the drag was performed, but the habitat on either side of the dragged habitat was also recorded to characterize edge type. Edge drag habitats could be found in between 1) impervious and forest, 2) maintained grass and forest, or 3) maintained grass and water environments. [file 12889_2022_13989_MOESM3_ESM.pdf]
