# Supplementary material for: Integrating tick density and park visitor behaviors to assess the risk of tick exposure in urban parks on Staten Island, New York
Source: BMC Public Health. 2022 Aug 23;22:1602. doi: 10.1186/s12889-022-13989-x (PMC9396585; doi:10.1186/s12889-022-13989-x)
Supplement: Supplementary file 4 — Additional file 4. List of plant species found in unmaintained herbaceous and leaf litter habitats. [file 12889_2022_13989_MOESM4_ESM.pdf]

Additional File 4. List of plant species found in unmaintained herbaceous and leaf litter habitats.

Habitat	Common name	Species name
**Unmaintained Herbaceous**	Multiflora rose	*Rosa multiflora*
	Poison ivy	*Toxicodendron radicans*
	Japanese stilt grass	*Microstegium vimineum*
	Jewelweed	*Impatiens capensis*
	Mugwort	*Artemisia vulgaris*
	Japanese honeysuckle	*Lonicera spp.*
	Porcelain berry	*Ampelopsis brevipedunculata*
	Japanese wineberry	*Rubus phoenicolasius*
	Virginia creeper	*Parthenocissus quinquefoli*
	Smartweed	*Persicaria lapathifolia*
	Japanese knotweed	*Reynoutria japonica*
	Blackberry	*Rubus spp.*
	Ragweed	*Ambrosia spp.*
	Goldenrod	*Solidago spp.*
	Milkweed	*Asclepias syriacasedge*
	Nettle	*Urtica dioica*
	Wild sarsaparilla	*Aralia nudicaulis*
**Leaf Litter**	Oak	*Quercus spp.*
	Red Oak	*Quercus rubra*
	Beech	*Fagus grandifolia*
	Maple	*Acer spp.*
	Pine	*Pinus spp.*
