# Supplementary material for: Integrating tick density and park visitor behaviors to assess the risk of tick exposure in urban parks on Staten Island, New York
Source: BMC Public Health. 2022 Aug 23;22:1602. doi: 10.1186/s12889-022-13989-x (PMC9396585; doi:10.1186/s12889-022-13989-x)

Additional File 5. Example map of park visitor movement within an open space. Human movement was mapped using arrows to denote the directional movement of each individual. Elapsed time was noted when visitors remained in one location. All daily time intervals for one site are represented: red= 9am-12pm, blue=12pm-3pm, yellow=3pm-6pm.

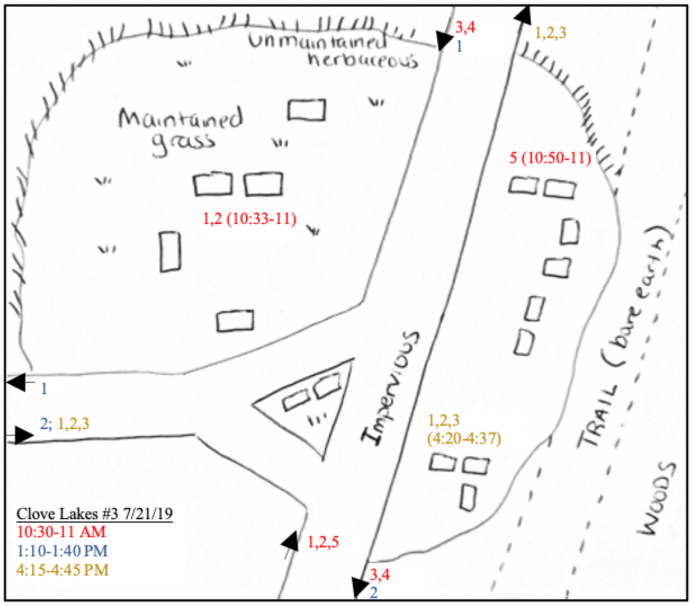

Supplement: Supplementary file 5 — Additional file 5. Example map of park visitor movement within an open space. Human movement was mapped using arrows to denote the directional movement of each individual. Elapsed time was noted when visitors remained in one location. All daily time intervals for one site are represented: red = 9 am-12 pm, blue = 12 pm-3 pm, yellow = 3 pm-6 pm. [file 12889_2022_13989_MOESM5_ESM.pdf]
