# Supplementary material for: Integrating tick density and park visitor behaviors to assess the risk of tick exposure in urban parks on Staten Island, New York
Source: BMC Public Health. 2022 Aug 23;22:1602. doi: 10.1186/s12889-022-13989-x (PMC9396585; doi:10.1186/s12889-022-13989-x)
Supplement: Supplementary file 6 — Additional file 6. KAP survey. [file 12889_2022_13989_MOESM6_ESM.pdf]

**Additional File 6.** KAP survey

**Questionnaire** (Answers will not be read aloud unless otherwise denoted)

1. Are you a Staten Island resident? Yes / No (Circle)

2. How often do you come here or use this space in general?
   - ☐ 1 = several times a year
   - ☐ 2 = once a month
   - ☐ 3 = once a week
   - ☐ 4 = almost every day
   - ☐ First Time

3. What activities to you often do in the parks?
   - ☐ Dog walk
   - ☐ Sleeping
   - ☐ Walk/Run
   - ☐ Read
   - ☐ Picnic
   - ☐ Sports
   - ☐ Others

4. Have you ever seen a tick? (Y/N)
5. Have you ever found a tick on you/ a member of your household? (Y/N)

6. Have you ever found a tick on your pet? (Y/N)

7. Which of these ticks have you seen?
   - ☐ 1
   - ☐ 2
   - ☐ 3
   - ☐ 4
   - ☐ 5
   - ☐ 6
   - ☐ 7
   - ☐ 8
   - ☐ None
   - ☐ I don't know

8. Where do you think people are being exposed to ticks here on Staten Island?
   - ☐ Parks/ Natural areas
   - ☐ Their own yard
   - ☐ Someone else's yard
   - ☐ Woods
   - ☐ Grassy areas

- ☐ Trails
- ☐ Other:

9. Have you ever avoided or stopped doing any activities because of the fear of tick exposure? If so, what activities?

10. How serious a problem are tick-transmitted diseases on Staten Island on a scale of 1 to 5 with 1 being not at all serious and 5 being extremely serious, in relation to other health concerns you might have (read the options)
    - ☐ 5- extremely serious
    - ☐ 4- very serious
    - ☐ 3- somewhat serious
    - ☐ 2- Slightly serious
    - ☐ 1- Not at all serious
    - ☐ Not sure

11. How many people do you -including yourself- who has had Lyme disease?

- ☐ None
- ☐ Do they live on Staten Island?
    - o Person 1 (Y/N)
    - o Person 2 (Y/N)
    - o Person 3 (Y/N)
- ☐ Are they a member of your household?
    - o Person 1 (Y/N)
    - o Person 2 (Y/N)
    - o Person 3 (Y/N)

12. On a scale of 1-5, where 5 is very likely, how likely is it that you would encounter a tick on yourself or a member of your household this summer? (**Read options)**
    - ☐ 5- very likely (over 80% chance)
    - ☐ 4- Somewhat likely (between 51-80%)
    - ☐ 3- Equally likely/unlikely (50/50%)
    - ☐ 2-Somewhat unlikely (between 10-49% chance)
    - ☐ 1- Very unlikely (less than 10% chance)
    - ☐ Not sure

13. Can you tell me how ticks get infected with the Lyme bacteria?
    - ☐ Don't know
    - ☐ They all have it
    - ☐ By feeding on mice/ other small mammals
    - ☐ By feeding on deer
    - ☐ By feeding on an infected person
    - ☐ Other

14. What tick prevention methods have you heard of?
    - ☐ Repellent

- Wearing light colored clothing
- Avoiding tick habitats
- Wearing long sleeves
- Tucking pants in socks
- Bathing after outdoor activities
- Vaccination
- Other
- None
- Not sure

15. How do ***you*** protect yourself from tick bites?
    - Check for ticks after being outdoors
    - Repellent
    - Wearing light colored clothing
    - Avoiding tick habitats
    - Wearing long sleeves
    - Tucking pants in socks
    - Bathing after outdoor activities
    - Vaccination
    - None
    - Not sure
    - Other

16. What would you do if you found a tick on yourself or a member of your household (as soon as you found one and after had found one)?
    - Remove it

    If removed, how?
    - Apply Vaseline
    - Check for rash then see a doctor
    - Send the tick for testing
    - See a doctor/vet right away?
    - Report it on the tick app
    - Nothing
    - Don't know
    - Other
17. How often do you check for ticks after being outdoors? (read answers)
    - Never
    - Some of the time
    - Every time outdoors

18. What would you say are the main reasons for skipping tick checks after being outdoors?
    - Forget
    - Don't have time
    - Laziness (don't want to)

- Seasonal importance
- Type of activity
- It’s not important
- Not in an area with ticks
- I don’t know how they look like/what to look for
- Other

19. How often do you use tick/insect repellent?
    - Never (1)
    - Some of the time (2)
    - Every time outdoors (3)
    - If so, what kind?

20. What is your main source of information about tick-borne diseases?
    - School
    - Friend
    - Family
    - Vet
    - TV/radio
    - Internet
    - Other

21. What do you think can be done on Staten Island to reduce the cases of Lyme disease?
    - Sterilize deer
    - Spray pesticides in natural area/parks
    - Spray pesticides in people’s yards
    - Kill mice
    - Kill ticks on mice/deer
    - Nothing
    - Education
    - Monitoring
    - It’s a personal matter (use of personal protective measures)
    - Other

**Demographics**

22. Age:

23. Gender:

24. How would you classify yourself in regards to race?

25. Do you consider yourself Hispanic or Latino? (Y/N)

26. What is the highest level of education you have completed?

**Observational (not asked):**

27. Respondent activity:
    a. Socializing
    b. Eating
    c. Reading print material
    d. Using phone
    e. Reading
    f. Sitting on bench
    g. Walking
    h. With dog
    i. With children
    j. Other:
