# Supplementary material for: Integrating tick density and park visitor behaviors to assess the risk of tick exposure in urban parks on Staten Island, New York
Source: BMC Public Health. 2022 Aug 23;22:1602. doi: 10.1186/s12889-022-13989-x (PMC9396585; doi:10.1186/s12889-022-13989-x)

Additional File 7. Arthropod samples including ticks and non-tick specimens. Respondents were asked to distinguish which were ticks given 1) Eastern ash bark beetle, 2) American dog tick adult, 3) swallow bug, 4) drugstore bug, 5) lone star tick adult, 6) deer tick adult, 7) flea, and 8) deer tick nymph.

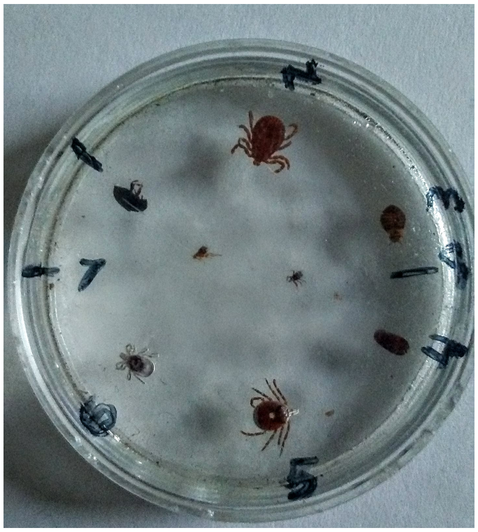

Supplement: Supplementary file 7 — Additional file 7. Arthropod samples including ticks and non-tick specimens. Respondents were asked to distinguish which were ticks given 1) Eastern ash bark beetle, 2) American dog tick adult, 3) swallow bug, 4) drugstore bug, 5) lone star tick adult, 6) deer tick adult, 7) flea, and 8) deer tick nymph. [file 12889_2022_13989_MOESM7_ESM.pdf]
