# Supplementary material for: Integrating tick density and park visitor behaviors to assess the risk of tick exposure in urban parks on Staten Island, New York
Source: BMC Public Health. 2022 Aug 23;22:1602. doi: 10.1186/s12889-022-13989-x (PMC9396585; doi:10.1186/s12889-022-13989-x)
Supplement: Supplementary file 8 — Additional file 8. Counts (n) and density (d) per 100 m2 of tick larvae (L), nymphs (N), and adults (A) in each park and site. [file 12889_2022_13989_MOESM8_ESM.pdf]

**Additional File 8.** Counts (n) and density (d) per 100 m$^2$ of tick larvae (L), nymphs (N), and adults (A) in each park and site.

| **Park** | **Site** | **Drag distance (m$^2$)** | ***A. americanum* n (d)** | | | ***H. longicornis* n (d)** | | | ***I. scapularis* n(d)** | | |
|---|---|---|---|---|---|---|---|---|---|---|---|
| | | | **L** | **N** | **A** | **L** | **N** | **A** | **L** | **N** | **A** |
| **Clove Lakes** | Open Space 1 | 4,063 | 0 | 3 (0) | 0 | 0 | 0 | 0 | 0 | 1 (0) | 0 |
| | Open Space 2 | 2,955 | 0 | 0 | 1 (0) | 0 | 0 | 0 | 0 | 0 | 0 |
| | Open Space 3 | 3,249 | 0 | 0 | 0 | 0 | 0 | 0 | 0 | 11 (0) | 0 |
| | Trail 1 | 1,774 | 0 | 0 | 0 | 0 | 0 | 0 | 0 | 3 (0) | 0 |
| | Trail 2 | 1,349 | 0 | 0 | 0 | 0 | 0 | 0 | 0 | 0 | 0 |
| | Trail 3 | 2,035 | 0 | 0 | 0 | 0 | 0 | 0 | 0 | 2 (0) | 0 |
| **Conference House** | Open Space 1 | 5,411 | 270 (5) | 39 (1) | 12 (0) | 1348 (25) | 802 (15) | 3 | 12 (0) | 27 (0) | 0 |
| | Open Space 2 | 2,762 | 14 (1) | 6 (0) | 7 (0) | 50 (2) | 117 (4) | 1 | 3 (0) | 1 (0) | 0 |
| | Trail 1 | 3,398 | 839 (25) | 55 (2) | 3 (0) | 2485 (73) | 839 (25) | 8 | 42 (1) | 24 (1) | 0 |
| | Trail 2 | 3,238 | 479 (15) | 50 (2) | 3 (0) | 162 (5) | 841 (26) | 1 | 3 (0) | 7 (0) | 0 |
| **Willowbrook** | Open Space 1 | 3,100 | 52 (2) | 1 (0) | 0 | 0 | 0 | 0 | 3 (0) | 1 (0) | 0 |
| | Open Space 2 | 3,687 | 133 (4) | 2 (0) | 0 | 0 | 0 | 0 | 782 (21) | 1 (0) | 0 |
| | Trail 1 | 238 | 0 | 0 | 0 | 0 | 0 | 0 | 1 (0) | 2 (1) | 0 |
| | Trail 2 | 3,316 | 0 | 1 (0) | 2 (0) | 0 | 0 | 0 | 0 | 5 (0) | 0 |
