# Supplementary material for: Integrating tick density and park visitor behaviors to assess the risk of tick exposure in urban parks on Staten Island, New York
Source: BMC Public Health. 2022 Aug 23;22:1602. doi: 10.1186/s12889-022-13989-x (PMC9396585; doi:10.1186/s12889-022-13989-x)

Additional File 9. Tick phenology in Staten Island parks. The proportional activity of each life stage from late- May to mid-August for *A. americanum, I. scapularis* and *H. longicornis* are shown.

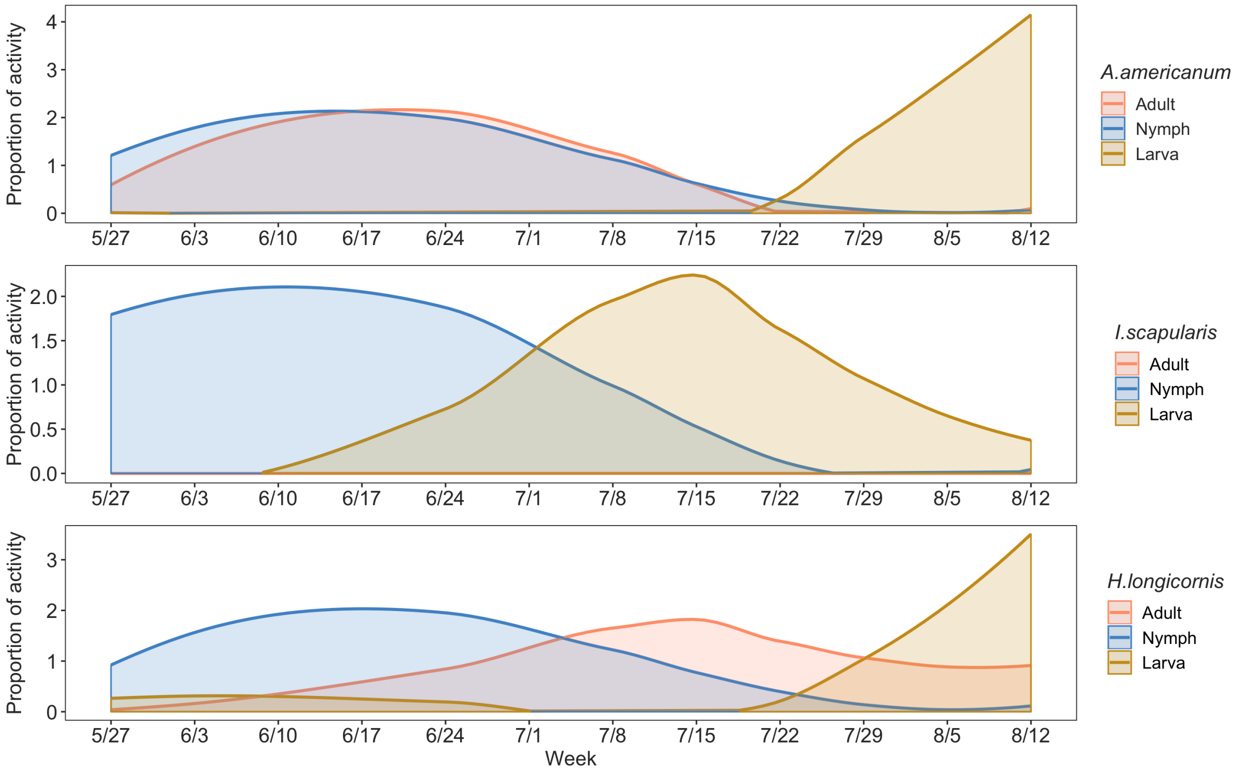

Supplement: Supplementary file 9 — Additional file 9. Tick phenology in Staten Island parks. The proportional activity of each life stage from late- May to mid-August for A. americanum, I. scapularis and H. longicornis are shown. [file 12889_2022_13989_MOESM9_ESM.pdf]
