# Supplementary material for: Integrating tick density and park visitor behaviors to assess the risk of tick exposure in urban parks on Staten Island, New York
Source: BMC Public Health. 2022 Aug 23;22:1602. doi: 10.1186/s12889-022-13989-x (PMC9396585; doi:10.1186/s12889-022-13989-x)
Supplement: Supplementary file 10 — Additional file 10. Nymph counts (n) and density per 100 m2 (d) by site type and park. Open spaces had either an edge (E) or no edge (NE). [file 12889_2022_13989_MOESM10_ESM.pdf]

Additional File 10. Nymph counts (n) and density per 100 m^2 (d) by site type and park. Open spaces had either an edge (E) or no edge (NE).

Park	Site type	Total distance (m^2)	*A. americanum* n (d)	*H. longicornis* n (d)	*I. scapularis* n (d)
**Clove Lakes**	Open space NE	5,385	1 (0.02)	0	7 (0.13)
	Open space E	4,741	2 (0.04)	0	5 (0.11)
	Trail	6,040	0	0	5 (0.08)
**Conference House**	Open space NE	4,248	8 (0.19)	79 (1.86)	0
	Open space E	1,043	0	70 (6.71)	1 (0.10)
	Trail	9,518	142 (1.49)	2,450 (25.74)	58 (0.61)
**Willowbrook**	Open space NE	3,777	2 (0.05)	0	1 (0.03)
	Open space E	3,010	1 (0.03)	0	1 (0.03)
	Trail	5,698	1 (0.02)	0	7 (0.12)
