# Supplementary material for: Integrating tick density and park visitor behaviors to assess the risk of tick exposure in urban parks on Staten Island, New York
Source: BMC Public Health. 2022 Aug 23;22:1602. doi: 10.1186/s12889-022-13989-x (PMC9396585; doi:10.1186/s12889-022-13989-x)
Supplement: Supplementary file 11 — Additional file 11. Nymph counts (n) and density per 100 m2 (d) by drag habitat and park. [file 12889_2022_13989_MOESM11_ESM.pdf]

Additional File 11. Nymph counts (n) and density per 100 m^2 (d) by drag habitat and park.

**Park**	**Drag habitat**	**Total distance (m^2)**	***A. americanum* n (d)**	***H. longicornis* n (d)**	***I. scapularis* n (d)**
**Clove Lakes**	Leaf litter	5,873	1 (0.02)	0	10 (0.17)
	Maintained grass	3,218	0	0	0
	Unmaintained herbaceous	7,075	2 (0.03)	0	7 (0.10)
**Conference House**	Leaf litter	NA	NA	NA	NA
	Maintained grass	3,914	7 (0.18)	43 (1.10)	0
	Unmaintained herbaceous	10,895	143 (1.31)	2,556 (23.46)	59 (0.54)
**Willowbrook**	Leaf litter	4,715	2 (0.04)	0	6 (0.13)
	Maintained grass	3,346	1 (0.03)	0	1 (0.03)
	Unmaintained herbaceous	4,424	1 (0.02)	0	2 (0.05)
