# Supplementary material for: Integrating tick density and park visitor behaviors to assess the risk of tick exposure in urban parks on Staten Island, New York
Source: BMC Public Health. 2022 Aug 23;22:1602. doi: 10.1186/s12889-022-13989-x (PMC9396585; doi:10.1186/s12889-022-13989-x)
Supplement: Supplementary file 12 — Additional file 12. Counts of park visitors by age group and gender in each site type and habitat. The total number of unique visitors (n) and within-group percentage (%) of visitors in each site and habitat. Habitats include impervious (I), maintained grass (MG), leaf litter (LL), and unmaintained herbaceous (UH), with LL and UH being the most hazardous for tick encounter. NA denotes that the habitat type was not present in the park. Hyphenated habitats indicate that a visitor passed through two different habitats in a single movement event. Visitors may be recorded multiple times if they spent time in multiple habitats during their visit. [file 12889_2022_13989_MOESM12_ESM.pdf]

Additional File 12. Counts of park visitors by age group and gender in each site type and habitat. The total number of unique visitors (n) and within-group percentage (%) of visitors in each site and habitat. Habitats include impervious (I), maintained grass (MG), leaf litter (LL), and unmaintained herbaceous (UH), with LL and UH being the most hazardous for tick encounter. NA denotes that the habitat type was not present in the park. Hyphenated habitats indicate that a visitor passed through two different habitats in a single movement event. Visitors may be recorded multiple times if they spent time in multiple habitats during their visit.

**Park**			**Site type n (%)**		**Habitat* n (%)**								
			Open space	Trail	I	I-MG	MG	MG-UH	UH	I-UH	LL	I-LL	MG-LL
**Clove Lakes**	**Age Group**												
		Child	194 (97)	6 (3)	160 (80)	33 (16.5)	31 (15.5)	1 (0.5)	6 (3)	NA	0 (0)	0 (0)	NA
		Teen	196 (94.7)	11 (5.3)	170 (82.1)	39 (18.8)	11 (5.3)	0	10 (4.8)	NA	1 (0.5)	0	NA
		Adult	179 (95.4)	86 (4.5)	1656 (88.2)	198 (10.5)	112 (6.5)	1 (0.05)	44 (2.3)	NA	42 (2.2)	2 (0.1)	NA
		Senior	468 (95.5)	22 (4.5)	432 (88.2)	47 (9.6)	20 (4.1)	1 (0.2)	13 (2.7)	NA	9 (1.8)	1 (0.2)	NA
	**Gender**												
		Female	1148 (96)	46 (3.9)	1051 (82)	110 (8.5)	73 (5.6)	1 (<1)	28 (2.2)	NA	18 (1.4)	0 (0)	NA
		Male	1501 (94.8)	82 (5.2)	1367 (73.4)	207 (11.1)	202 (10.8)	2 (0.1)	46 (2.5)	NA	36 (1.9)	2 (0.1)	NA
**Conference House**	**Age Group**												
		Child	268 (93.1)	20 (6.9)	233 (80.9)	46 (16)	28 (9.7)	0 (0)	22 (7.6)	1 (<1)	NA	NA	NA
		Teen	121 (80.1)	30 (19.9)	85 (56.3)	35 (23.2)	34 (22.5)	2 (1.3)	34 (22.5)	0 (0)	NA	NA	NA
		Adult	438 (75.3)	144 (24.7)	271 (46.6)	132 (22.7)	102 (17.5)	18 (3.1)	149 (25.6)	13 (2.2)	NA	NA	NA

	Senior	108 (65)	58 (34.9)	56 (33.7)	36 (21.7)	24 (14.5)	3 (1.8)	66 (39.8)	8 (4.8)	NA	NA	NA
	**Gender**											
	Female	451 (85.3)	78 (14.7)	339 (64)	108 (20.4)	83 (15.7)	9 (1.7)	83 (15.7)	7 (1.3)	NA	NA	NA
	Male	475 (73.1)	174 (26.9)	302 (46.6)	142 (21.9)	105 (16.2)	14 (2.1)	188 (29)	15 (2.2)	NA	NA	NA
**Willowbrook**	**Age Group**											
	Child	347 (86.3)	55 (13.7)	241 (60)	33 (8.2)	202 (50.2)	NA	50 (12.4)	NA	4 (0.01)	NA	19 (4.7)
	Teen	105 (60.3)	69 (39.7)	65 (37.4)	3 (1.7)	44 (25.3)	NA	69 (39.7)	NA	2 (1.1)	NA	2 (1.1)
	Adult	859 (72.4)	328 (27.6)	474 (39.9)	66 (5.6)	363 (30.6	NA	284 (23.9)	NA	50 (42.1)	NA	22 (1.9)
	Senior	152 (71)	62 (29)	107 (0.5)	12 (5.6)	35 (16.4)	NA	57 (26.6)	NA	6 (2.8)	NA	4 (1.9)
	**Gender**											
	Female	703 (74.4)	242 (25.6)	411 (36.6)	66 (5.9)	390 (34.8)	NA	221 (19.7)	NA	23 (2)	NA	11 (0.9)
	Male	760 (73.6)	272 (26.4)	476 (38.9)	49 (4)	384 (31.4)	NA	240 (19.6)	NA	39 (3.2)	NA	36 (2.9)
