# Supplementary material for: Integrating tick density and park visitor behaviors to assess the risk of tick exposure in urban parks on Staten Island, New York
Source: BMC Public Health. 2022 Aug 23;22:1602. doi: 10.1186/s12889-022-13989-x (PMC9396585; doi:10.1186/s12889-022-13989-x)
Supplement: Supplementary file 13 — Additional file 13. The counts of visitors (n) and average time spent (minutes) in each habitat by age group and gender. Habitats include impervious (I), leaf litter (LL), maintained grass (MG), and unmaintained herbaceous (UH). Only data from open spaces is represented since elapsed time on trails could not be captured, and NA denotes that the habitat type was not present in the park open spaces. Hyphenated habitats indicate that a visitor passed through two different habitats in a single movement event. Visitors may be recorded multiple times if they spent time in multiple habitats during their visit. [file 12889_2022_13989_MOESM13_ESM.pdf]

Additional File 13. The counts of visitors (n) and average time spent (minutes) in each habitat by age group and gender. Habitats include impervious (I), leaf litter (LL), maintained grass (MG), and unmaintained herbaceous (UH). Only data from open spaces is represented since elapsed time on trails could not be captured, and NA denotes that the habitat type was not present in the park open spaces. Hyphenated habitats indicate that a visitor passed through two different habitats in a single movement event. Visitors may be recorded multiple times if they spent time in multiple habitats during their visit.

**Park**			**Habitat n (avg min)**								
			I	I-MG	MG	MG-UH	UH	I-UH	LL	I-LL	MG-LL
**Clove Lakes**	**Age group**										
		Child	160 (6)	33 (0.8)	31 (15.4)	1 (0.1)	NA	NA	NA	0 (0)	NA
		Teen	170 (3)	39 (1)	11 (7.5)	0 (0)	NA	NA	NA	0 (0)	NA
		Adult	1653 (2.6)	198 (1.2)	112 (5.2)	1 (0.1)	NA	NA	NA	2 (0.1)	NA
		Senior	429 (2)	47 (1.3)	20 (2.5)	1 (3)	NA	NA	NA	0 (0)	NA
	**Gender**										
		Female	1049 (2.6)	110 (1)	73 (9.6)	1 (0.1)	NA	NA	NA	0 (0)	NA
		Male	1363 (3)	207 (1.2)	101 (4.8)	2 (1.5)	NA	NA	NA	2 (0.1)	NA
**Conference House**	**Age group**										
		Child	27 (5.7)	44 (1.1)	23 (12.4)	0 (0)	1 (3)	1 (0.1)	NA	NA	NA
		Teen	6 (0.9)	35 (1.3)	33 (8.1)	2 (0.1)	4 (0.1)	0 (0)	NA	NA	NA
		Adult	80 (3.6)	131 (1.7)	100 (8.8)	18 (2.1)	5 (1.4)	13 (0.6)	NA	NA	NA
		Senior	42 (8.4)	36 (1.2)	23 (7.2)	3 (0.2)	7 (0.1)	8 (0.1)	NA	NA	NA
	**Gender**										
		Female	85 (5.3)	106 (1.4)	81 (9.2)	9 (2.8)	5 (1.4)	7 (0.5)	NA	NA	NA
		Male	70 (4.6)	141 (1.5)	98 (8.5)	14 (0.9)	12 (0.3)	15 (0.3)	NA	NA	NA
**Willowbrook**	**Age group**										
		Child	235 (7.6)	33 (0.8)	238 (4.5)	NA	NA	NA	0 (0)	NA	19 (4)

	Teen	65 (3.3)	3 (1)	60 (9.1)	NA	NA	NA	2 (0.1)	NA	2 (2.5)
	Adult	465 (3.2)	66 (1.4)	433 (8.1)	NA	NA	NA	6 (1)	NA	22 (3.8)
	Senior	104 (2.4)	12 (1.2)	42 (13.2)	NA	NA	NA	1 (0.1)	NA	4 (8.2)
**Gender**										
	Female	401 (4.8)	66 (1.4)	389 (6.9)	NA	NA	NA	2 (1)	NA	11 (2)
	Male	468 (3.7)	49 (1)	384 (7.6)	NA	NA	NA	7 (0.6)	NA	36 (4.9)
