# Supplementary material for: Integrating tick density and park visitor behaviors to assess the risk of tick exposure in urban parks on Staten Island, New York
Source: BMC Public Health. 2022 Aug 23;22:1602. doi: 10.1186/s12889-022-13989-x (PMC9396585; doi:10.1186/s12889-022-13989-x)
Supplement: Supplementary file 14 — Additional file 14. Counts of visitors (n) engaging in an activity and average elapsed time (min) spent in each park habitat. Information includes only includes open space site types because time spent could not be captured for individuals in trails. Individuals may be recorded multiple times if their activities and habitat usage changed during their visit in the site. [file 12889_2022_13989_MOESM14_ESM.pdf]

Additional File 14. Counts of visitors (n) engaging in an activity and average elapsed time (min) spent in each park habitat. Information includes only includes open space site types because time spent could not be captured for individuals in trails. Individuals may be recorded multiple times if their activities and habitat usage changed during their visit in the site.

Park	Habitat	Activity	n	min
**Clove Lakes**	**Impervious**	Walking	1711	1.4
		Jogging/running	396	0.6
		Biking	117	0.5
		Picnicking	94	21.8
		Exercising	85	6.8
		Standing	84	6.8
		Sitting	83	9.6
		Fishing	53	22.4
		Other transport	21	1.8
		Socializing	4	18.5
		Working	4	6.3
		Playing	4	2.4
		Birding	3	3.0
		Reading	2	20.0
		Arts	2	3.0
		Sports	1	30.0
	**Impervious- leaf litter**	Walking	2	0.1
	**Impervious- maintained grass**	Walking	289	1.0
		Jogging/running	11	0.8
		Other transport	8	0.5
		Exercising	6	10.3
		Biking	2	0.7
		Standing	2	0.6
		Picnicking	2	0.1
		Birding	1	11.0
		Working	1	9.0
	**Maintained grass**	Picnicking	55	18.8
		Walking	54	0.7
		Standing	50	1.7
		Sitting	40	12.7
		Other transport	6	0.8
		Exercising	5	17.0

		Sports	3	11.3
		Tanning	2	10.5
		Working	2	4.0
		Birding	1	4.0
	**Maintained grass-unmaintained herbaceous**	Jogging/running	2	0.1
		Walking	1	3.0
**Conference House**	**Impervious**	Walking	95	0.7
		Touring	35	4.8
		Standing	20	5.7
		Sitting	15	7.3
		Photography	14	24.9
		Gardening	13	22.1
		Socializing	6	13.5
		Biking	5	0.2
		Arts	3	3.5
		Other transport	2	2.6
	**Impervious-maintained grass**	Walking	175	0.9
		Touring	37	0.1
		Arts	14	14.0
		Biking	9	0.4
		Sitting	3	3.3
		Photography	2	1.1
		Working	2	0.3
		Sports	2	0.1
		Jogging/running	1	0.1
		Tanning	1	0.1
	**Impervious-unmaintained herbaceous**	Walking	18	0.5
		Biking	3	0.1
		Jogging/running	1	0.1
	**Maintained grass**	Sitting	55	14.2
		Walking	53	1.9
		Picnicking	34	16.5
		Touring	21	7.6
		Standing	20	6.3
		Sports	13	7.7
		Working	6	3.2
		Tanning	5	20.0
		Photography	5	5.8
		Biking	4	1.8

		Arts	3	25.0
		Playing	2	10.0
		Exercising	1	2.0
	**Maintained grass-unmaintained herbaceous**	Walking	20	1.6
		Working	1	3.0
		Other transport	1	1.0
		Biking	1	0.1
	**Unmaintained herbaceous**	Walking	16	0.8
		Touring	1	0.1
**Willowbrook**	**Impervious**	Walking	516	1.3
		Playing	273	11.3
		Biking	44	0.6
		Sitting	31	12.7
		Jogging/running	25	0.4
		Other transport	12	0.7
		Standing	8	4.2
		Reading	1	30.0
		Picnicking	1	1.0
	**Impervious-maintained grass**	Walking	110	1.3
		Standing	4	1.0
		Playing	3	1.0
		Other transport	1	1.1
		Biking	1	1.0
	**Leaf litter**	Walking	8	0.7
		Biking	1	1.0
	**Maintained grass**	Walking	536	0.7
		Picnicking	434	24.7
		Socializing	287	28.1
		Sitting	154	13.0
		Standing	106	5.0
		Playing	30	9.2
		Sports	9	12.3
		Jogging/running	8	0.4
		Other transport	5	6.4
		Working	5	2.8
		Exercising	2	29.0
		Tanning	1	8.0
		Arts	1	4.3
	**Maintained grass-leaf litter**	Walking	32	2.8
		Playing	8	13.6

	Standing	4	1.5
	Jogging/running	4	0.6
	Sitting	2	2.5
	Picnicking	1	30.0
