# Supplementary material for: Integrating tick density and park visitor behaviors to assess the risk of tick exposure in urban parks on Staten Island, New York
Source: BMC Public Health. 2022 Aug 23;22:1602. doi: 10.1186/s12889-022-13989-x (PMC9396585; doi:10.1186/s12889-022-13989-x)
Supplement: Supplementary file 15 — Additional file 15. KAP responses. Asterisk denotes multiple responses allowed. [file 12889_2022_13989_MOESM15_ESM.pdf]

Additional File 15. KAP responses. Asterisk denotes multiple responses allowed.

Question	Answer	Number of respondents	Response n (%)	Coded
**Background**				
**Visitation frequency**	Several times a year	190	31 (16.3)	1
	Once a month	190	20 (10.5)	2
	Once a week	190	19 (10)	3
	Several times a week	190	35 (18.4)	4
	Almost everyday	190	69 (36.3)	5
	First time	190	13 (6.8)	6
	Other	190	3 (1.6)	0
**Park activities***	Dog walk	190	39 (20.5)	0/1
	Walk/ run/ hike	190	102 (53.7)	0/1
	Read	190	6 (3.2)	0/1
	Picnic	190	8 (4.2)	0/1
	Sports	190	16 (8.4)	0/1
	Relax	190	28 (14.7)	0/1
	Play	190	15 (7.9)	0/1
	Fish	190	10 (5.3)	0/1
	Bike	190	12 (6.3)	0/1
	Work	190	14 (7.4)	0/1
	Park events	190	13 (6.8)	0/1
	Art	190	11 (5.8)	0/1
	Watch wildlife	190	13 (6.8)	0/1
	Other	190	20 (10.5)	0/1
**Have you seen a tick?**	Yes	190	114 (60)	0/1
**Have you found a tick on you or household member?**	Yes	114	61 (53.5)	0/1
**Have you found a tick on your pet?**	Yes	114	62 (54.4)	0/1
**Do you know anyone with Lyme?**	Yes	190	84 (44.2)	0/1
	One person	188	58 (30.9)	0/1
	Two people	185	23 (12.4)	0/1
	Three people	185	12 (6.5)	0/1
	More than three	185	13 (7)	0/1
	Person 1	105	77 (73.3)	0/1

**Does the person with Lyme disease live on Staten Island?**	Person 2	47	30 (63.8)	0/1
	Person 3	24	12 (50)	0/1
**Is the person with Lyme disease a member of your house?**	Person 1	104	19 (18.3)	0/1
	Person 2	48	2 (4.2)	0/1
	Person 3	25	0 (0)	0/1
**Knowledge**				
**Which of these are ticks?***	Eastern ash bark beetle	113	11 (9.7)	0/1
	American dog tick	113	72 (63.7)	0/1
	Swallow bug	113	8 (7.1)	0/1
	Drugstore bug	113	3 (2.7)	0/1
	Lone star tick	113	43 (38.1)	0/1
	Deer tick adult	113	30 (26.5)	0/1
	Flea	113	5 (4.4)	0/1
	Deer tick nymph	113	4 (3.5)	0/1
	None	113	3 (2.7)	0/1
	I don't know	113	16 (14.2)	0/1
**Where are people getting exposed to ticks?***	Parks	190	82 (43.2)	0/1
	Yards	190	12 (6.3)	0/1
	Woods	190	52 (27.4)	0/1
	Grass	190	32 (16.8)	0/1
	Trails	190	7 (3.7)	0/1
	Deer areas	190	24 (12.6)	0/1
	Everywhere	190	10 (5.3)	0/1
	Water	190	6 (3.2)	0/1
	Other	190	14 (7.4)	0/1
	I don't know	190	22 (11.6)	0/1
**How do ticks get infected with Lyme disease?***	All are infected	190	3 (1.6)	0/1
	Mice	190	16 (8.4)	0/1
	Deer	190	43 (22.6)	0/1
	Infected animals	190	17 (8.9)	0/1
	Other	190	6 (3.2)	0/1
	I don't know	190	121 (63.7)	0/1
**Knowledge prevention methods***	Repellent	190	117 (61.6)	0/1
	Light colored clothing	190	15 (7.9)	0/1
	Avoid tick habitat	190	68 (35.8)	0/1
	Long sleeves	190	76 (40)	0/1

	Pants into socks	190	44 (23.2)	0/1
	Shower	190	10 (5.3)	0/1
	Vaccine	190	4 (2.1)	0/1
	Tick check	190	47 (24.7)	0/1
	Pet repellent	190	7 (3.7)	0/1
	Other	190	34 (17.9)	0/1
	None	190	33 (17.4)	0/1
**What would you do if you found a tick?***	Remove	189	136 (72)	0/1
	Remove: tweezers	136	70 (51.5)	0/1
	Remove: fingers	136	20 (14.7)	0/1
	Remove: burn	136	16 (11.8)	0/1
	Remove: kill	136	8 (5.9)	0/1
	Remove: Vaseline	136	9 (6.6)	0/1
	Send it for testing	189	8 (4.2)	0/1
	Go to doctor	189	52 (27.5)	0/1
	Other	189	16 (8.5)	0/1
	I don't know	189	4 (2.1)	0/1
**How to reduce Lyme disease on Staten Island?***	Spraying	186	65 (34.9)	0/1
	Education	186	55 (29.6)	0/1
	Deer control/reduction	186	33 (17.7)	0/1
	Other	186	20 (10.8)	0/1
	I don't know	186	19(10.2)	0/1
	Personal protection	186	15 (8.1)	0/1
	Vegetation management	186	12 (6.5)	0/1
	Nothing can be done	186	8 (4.3)	0/1
	Mice control	186	6 (3.2)	0/1
	Tick reduction	186	6 (3.2)	0/1
	Increase predators of ticks (e.g. opossums)	186	4 (2.2)	0/1 0/1
	Monitor/surveillance	186	2 (1.1)	0/1
**Attitudes**				
**How serious are tick-transmitted diseases on Staten Island?**	Not at all serious	190	14 (7.4)	0
	Slightly serious	190	16 (8.4)	1
	Somewhat serious	190	42 (22.1)	2
	Very serious	190	33 (17.4)	3
	Extremely serious	190	47 (24.7)	4
	Not sure	190	38 (20)	2
	Cost	187	0 (0)	0/1

**Reasons for not using repellent?***	Worry	187	1 (0.5)	0/1
	Health	187	75 (40.1)	0/1
	Feel	187	12 (6.4)	0/1
	Ineffective	187	4 (2.1)	0/1
	Need	187	7 (3.7)	0/1
	None	187	90 (48.1)	0/1
	Other	187	6 (3.2)	0/1
**Perceived probability of tick encounter**	Very unlikely	189	82 (43.4)	0
	Somewhat unlikely	189	16 (8.5)	1
	Equally likely/unlikely	189	30 (15.9)	2
	Somewhat likely	189	13 (6.9)	3
	Very likely	189	33 (17.5)	4
	Not sure	189	15 (7.9)	2
**Reasons for not checking for ticks***	Forget	188	22 (11.7)	0/1
	No time	188	15 (8)	0/1
	Laziness	188	23 (12.2)	0/1
	Seasonal importance	188	2 (1.1)	0/1
	Activity dependent	188	5 (2.7)	0/1
	Not important	188	18 (9.6)	0/1
	Area dependent	188	46 (24.5)	0/1
	No previous experience with ticks	188	19 (10.1)	0/1
	Don't think to do it	188	19 (10.1)	0/1
	Negligence	188	7 (3.7)	0/1
	Ignorance	188	13 (6.9)	0/1
	I don't know	188	13 (6.9)	0/1
	Always checks	188	13 (6.9)	0/1
	Other	188	8 (4.3)	0/1
**Practices**				
**Changed activities because of ticks?**	Yes	190	48 (25.3)	0/1
**What personal protection methods do you use?***	Repellent	189	54 (28.6)	0/1
	Light colored clothing	189	7 (3.7)	0/1
	Avoid habitat	189	58 (30.7)	0/1
	Long sleeves	189	49 (25.9)	0/1
	Pants into socks or wearing long socks	189	26 (13.8)	0/1
	Shower	189	8 (4.2)	0/1
	Tick check	189	34 (18)	0/1

	Other	189	13 (6.9)	0/1
	None or I don't know	189	62 (32.8)	0/1
**Tick check frequency**	Never	188	79 (42)	1
	Some of the time	188	28 (14.9)	2
	All of the time	188	71 (37.8)	3
**Repellent use**	Never	186	83 (44.6)	1
	Some of the time	186	57 (30.6)	2
	All of the time	186	46 (24.7)	3
